# Green synthesis of glyco-CuInS_2_ QDs with visible/NIR dual emission for 3D multicellular tumor spheroid and in vivo imaging

**DOI:** 10.1186/s12951-023-01859-6

**Published:** 2023-04-01

**Authors:** Xiaolin Guan, Liyuan Zhang, Shoujun Lai, Jiaming Zhang, Jingyu Wei, Kang Wang, Wentao Zhang, Chenghao Li, Jinhui Tong, Ziqiang Lei

**Affiliations:** 1 Present Address: Key Laboratory of Eco-Environment-Related Polymer Materials Ministry of Education, Key Laboratory of Polymer Materials Ministry of Gansu Province, College of Chemistry and Chemical Engineering, Northwest Normal University, Lanzhou, 730070 Gansu People’s Republic of China; 2 College of Chemical Engineering, Lanzhou University of Arts and Science, Lanzhou, 730000 Gansu People’s Republic of China; 3 Key Laboratory of Traditional Chinese Medicine Prevention and Treatment, Gansu University of Traditional Chinese Medicine, Lanzhou, 730000 China

**Keywords:** Glyco-quantum dots, Visible/NIR emission, Green synthetic approach, Bioimaging, 3D multicellular tumor spheroids

## Abstract

Glyco-quantum dots (glyco-QDs) have attracted significant interest in bioimaging applications, notably in cancer imaging, because they effectively combine the glycocluster effect with the exceptional optical properties of QDs. The key challenge now lies in how to eliminate the high heavy metal toxicity originating from traditional toxic Cd-based QDs for in vivo bioimaging. Herein, we report an eco-friendly pathway to prepare nontoxic Cd-free glyco-QDs in water by the “direct” reaction of thiol-ending monosaccharides with metal salts precursors. The formation of glyco-CuInS_2_ QDs could be explained by a nucleation-growth mechanism following the LaMer model. As-prepared four glyco-CuInS_2_ QDs were water-soluble, monodispersed, spherical in shape and exhibited size range of 3.0–4.0 nm. They exhibited well-separated dual emission in the visible region (500–590 nm) and near-infrared range (~ 827 nm), which may be attributable to visible excitonic emission and near-infrared surface defect emission. Meanwhile, the cell imaging displayed the reversibly distinct dual-color (green and red) fluorescence in tumor cells (HeLa, A549, MKN-45) and excellent membrane-targeting properties of glyco-CuInS_2_ QDs based on their good biorecognition ability. Importantly, these QDs succeed in penetrating uniformly into the interior (the necrotic zone) of 3D multicellular tumor spheroids (MCTS) due to their high negative charge (zeta potential values ranging from − 23.9 to − 30.1 mV), which overcame the problem of poor penetration depth of existing QDs in in vitro spheroid models. So, confocal analysis confirmed their excellent ability to penetrate and label tumors. Thus, the successful application in in vivo bioimaging of these glyco-QDs verified that this design strategy is an effective, low cost and simple procedure for developing green nanoparticles as cheap and promising fluorescent bioprobes.

**Supplementary Information:**

The online version contains supplementary material available at 10.1186/s12951-023-01859-6.

## Introduction

Nanobiosensor have played a key role in the fast cancer diagnosis and in clinical medicine and gained a great deal of attention in the areas of biological sciences [1, 2]. Recently, glyconanoparticles (GNPs) have been of increased interest as biofunctional nanosensors, which effectively combine the unique chemico-physical and optical properties of various nanoparticles with the characteristics of the carbohydrate coating [3–5]. Carbohydrates on the surface of NPs not only enhance the water solubility, biocompatibility and stability, but also endow these NPs with recognition ability. Thus, the GNPs have shown great potential for biomedical applications, especially in cellular labeling and imaging [6, 7]. Therefore, research on the biological effects associated with carbohydrate and developing new GNPs have become a hot topic in recent years.

A variety of nanoparticles (NPs), including gold, iron oxide, and semi-conductor quantum dots (QDs), have been used as the carriers of carbohydrates to prepare GNPs since the first synthesis of carbohydrate-functionalized gold nanoparticles in 2001[8]. Among them, QDs have been found as an attractive biomedical resource and widely used in vitro and in vivo for multiple color imaging and targeted drug delivery due to their broad absorption, high quantum yield, and long-term photostability [9–11]. Besides, many researches indicate that QDs have great potential in cancer detection and treatment [12, 13]. Good water solubility, biocompatibility, and biological targeting of QDs, all of which are essential for biomedical application, could easily be achieved through surface modification or conjugation with carbohydrates. Therefore, glyco-quantum dots (glyco-QDs) have attracted significant attention and many different glyco-QDs have been developed by coating with a variety of carbohydrates. For example, Shin-Ichiro Nishimura et al. synthesized a series of carbohydrate-capped Cd-based quantum dots (CdSe/ZnS and CdSeTe/CdS) with a variety of common sugars, such as α-glucose, α-mannose, lactose, N-acetyl-β-D-glucosamine, and N-acetyl-β-D-galactosamine. In vivo near-infrared (NIR) fluorescence imaging of these glyco-QDs revealed the importance of the terminal sialic acid residues for achieving prolonged in vivo lifetime [14]. Recently, Richichi's group reported a kind of CdSe/ZnS QDs-based fluorescent glyconanoprobe as nanoprobes for carbonic anhydrase IX imaging in cancer cells [15]. However, to date, the majority of glyco-QDs related research in biomedicine focused on Cd-based QDs including CdS, CdSe, and CdTe QDs, which are often added by ZnS and/or ZnSe as protective shells. Unfortunately, many studies have identified that highly cytotoxic Cd^2+^ ions in QDs can been released in cellular environment, which would ultimately increase the cytotoxicity effect of these QDs in a biological environment [16, 17]. So, the broad potential applications of Cd-based QDs in clinical medicine have been questioned by many biomedical researchers due to the high toxicity of heavy metal cadmium [18]. Accordingly, the potential high toxicity of fluorescent GNPs based on Cd-based QDs severely hinders their biomedical application. These results motivated the development of Cd-free glyco-QDs with less toxicity.

Currently, there has been a growing effort to prepare environmentally friendly Cd-free and less toxic QDs with performance comparable to or even better than existing Cd-based QDs [19]. Among these Cd-free QDs, copper indium sulfide (CuInS_2_) QDs have shown the greatest application potential for biological applications. CuInS_2_ QDs are I-III-VI_2_ semiconductor nanocrystals and does not contain any toxic heavy metals. They can provide PL emission ranging from the visible to the NIR, and have broad absorption, size-tunable photoluminescence (PL), excellent optical, and chemical stability [20, 21]. However, the conventional synthesis of CuInS_2_ QDs usually employs toxic organic solvents and the hydrophobic QDs are not appropriate for their application in biomedical fields. In addition, the use of organic solvents in the synthetic process is harmful to environment. In the view of hydrophilicity, toxicity and environment protection, the aqueous synthesis of high quality CuInS_2_ QDs is desired. In recent years, many efforts have been devoted to the aqueous synthesis of CuInS_2_ QDs for biological applications. Therefore, CuInS_2_ QDs could offer the opportunity to realize the potential of glyco-QDs without the toxicity limitations in biological environment. However, there is little research on the preparation and application of GNPs materials based on CuInS_2_ QDs in the biomedical field [22].

In general, the synthetic protocols for the preparation of glyco-QDs reported so far can be mainly described as: covalent conjugation, thiolated-glycans exchange, adsorption, and biotin-streptavidin chemistry [23]. Among them, valence conjugation is generally regarded as a straightforward and simple method. By following this route, functional carbohydrates are directly covalently linked on the surface of QDs. For instance, Penadés et al. directly used the thiol-ending saccharides as capping ligand and stabilizer to form the GNPs based on CdS QDs in degassed water under room temperature by a straightforward single step method [24]. Obviously, the direct synthesis of high-quality semiconductor glyco-QDs in aqueous medium is less harmful to the environment and health of people compared with the conventional organic synthesis of QDs. And, the resultant QDs are better water-soluble and biocompatible. Nevertheless, to the best of our knowledge, there is almost no literature on the synthesis of glyco-QDs based on ternary CuInS_2_ QDs in aqueous solution. Therefore, there is a high need to develop nontoxic and water-soluble glyco-CuInS_2_ QDs capped by carbohydrates as bio-compatible labeling and imaging probes in various biological applications.

Therefore, we developed in this work a simple, green and reproducible methodology for the construction of glyco-QDs in water via direct synthesis methods. These target glyco-QDs were synthesized by the “direct” reaction of thiol-ending monosaccharides (SH-fructose, SH-galactose, SH-mannose, SH-glucose) with metal salts precursors (CuCl_2_·2H_2_O, InCl_3_·4H_2_O and CS(NH_2_)_2_) in water. The as-prepared glyco-QDs not only exhibited well-separated dual-wavelength emission in the visible light region (500 ~ 590 nm) and the NIR range (~ 827 nm) upon excitation spectral scan (215 nm to 515 nm), but also displayed the high photostablity, good water-solubility, favorable photoreversibility and noncytotoxicity. Moreover, these glyco-CuInS_2_ QDs exhibited excellent biocompatibility and good biorecognition ability. The investigation of cellular imaging in tumor cells (HeLa, A549, MKN-45) indicated their reversible dual-color (green and red) imaging function and excellent membrane-targeting properties originating from a large number of carbohydrates on the surface of QDs. More importantly, they could penetrate uniformly into the interior of three-dimensional (3D) multicellular tumor spheroids (MCTS) due to their high negative charge, which realized the deep penetration depth of QDs in in vitro spheroid models. In summary, these glyco-CuInS_2_ QDs have successfully achieved 2D and 3D spatiotemporal visualization of the cell membrane, thus providing a huge potential in the application of commercial probes for cell membrane imaging.

## Results and discussion

### Design and synthesis of four kinds of glycol-CuInS_2_ QDs

**Scheme 1:**
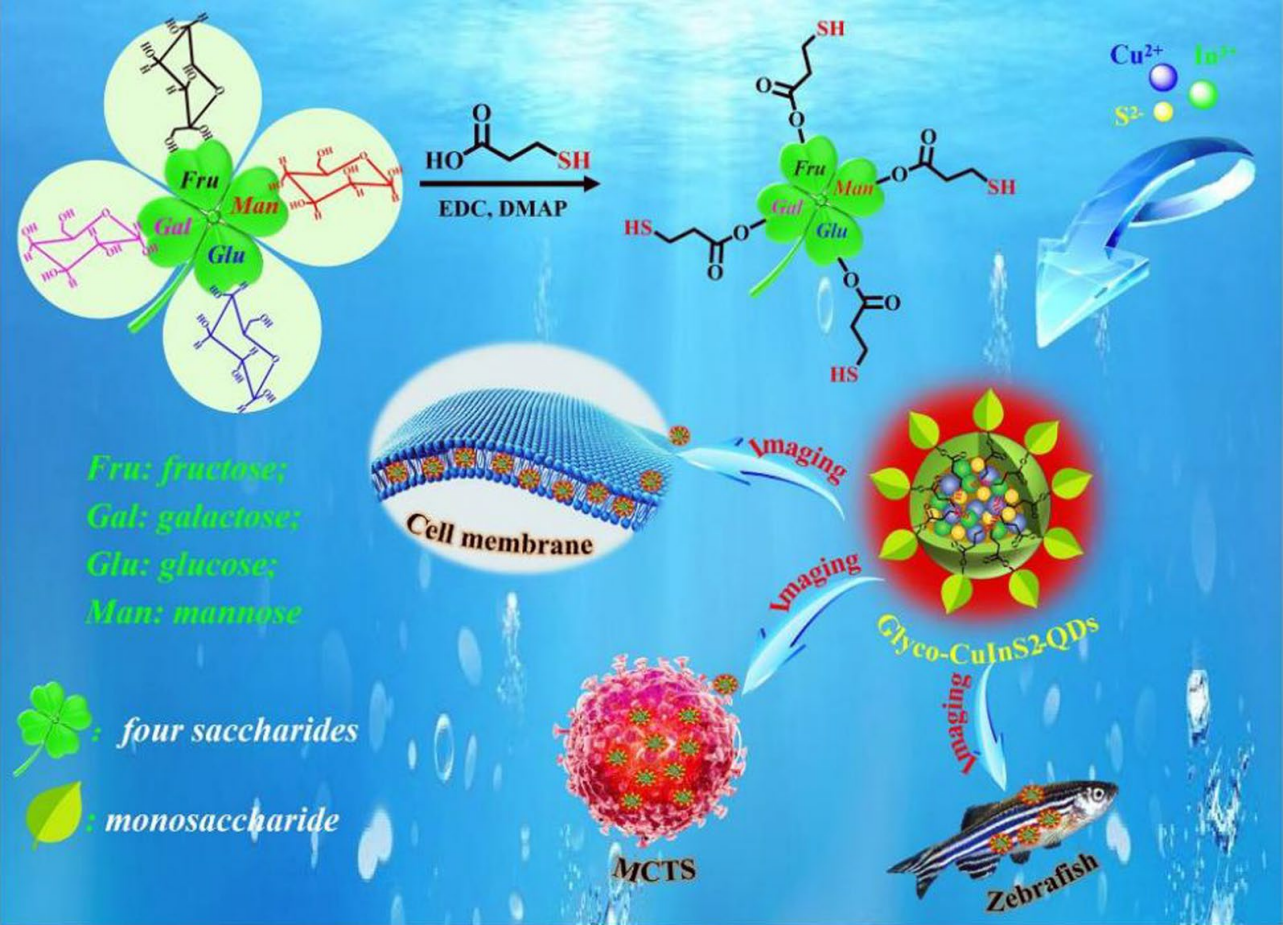
Schematic illustration of the synthesis and biolabelling process of four kinds of glycol-CuInS_2_ QDs

The most common synthetic protocols for the preparation of glyco-QDs are ligand exchange with hydrophilic QDs or surface modification with carbohydrate [25–27]. However, the former requires the use of toxic organic solvents and the other one undergoes complex multistep process. Therefore, toxic organic solvent-free technology and simple method for hydrophilic synthesis of QDs are desired. Thus, we proposed a simple, green and reproducible design methodology of the construction of low cost glyco-QDs by the “direct” reaction of neutral monosaccharides (D-fructose and D-glucose mainly from honey, D-galactose from animal milk and D-mannose from fruit peels) with metal salts precursors in aqueous media. Scheme 1 illustrates the synthetic routes to four kinds of glycol-CuInS_2_ QDs, and the detailed synthesis procedure are described in Additional Information. The thiolation of monosaccharide is a key intermediate in the total synthesis of glyco-CuInS_2_ QDs. As shown in Scheme 1, four thiol-functionalized monosaccharides (SH-fructose, SH-galactose, SH-mannose, SH-glucose) were obtained by using DCC/DMAP esterification procedure with 3-mercaptopropionic acid. Subsequently, four environmentally friendly glyco-CuInS_2_ QDs were synthesized by using only these thiol-functionalized monosaccharides as stabilizing agents and capping ligands in aqueous medium by reaction with CuCl_2_·2H_2_O, InCl_3_·4H_2_O and CS(NH_2_)_2_ under an Ar atmosphere.

In order to explore the optimal conditions for the synthesis of glyco-CuInS_2_ QDs, the molar ratios between Cu, In, S and the thiol-ending saccharide ligands, pH of the reaction solutions and reaction temperatures were systematically investigated. Additional file 1: Fig. S3–S5 show these changes in fluorescence intensity at 827 nm of the synthesized glyco-CuInS_2_ QDs at the different molar ratios, pH and reaction temperatures, respectively. Meanwhile, the optimal synthesis conditions are summarized in Additional file 1: Table S1. The experimental results show that the intensities of fluorescence of Fru-CuInS_2_, Gal-CuInS_2_, Man-CuInS_2_ and Glu-CuInS_2_ QDs reach the strongest when the ratios of Cu, In, S to ligands were 1:1:2:36, 1:1:2:24 or 1:1:2:12 at pH 11 ~ 12 and 150 °C, respectively. Moreover, the emission wavelength of these glyco-CuInS_2_ QDs do not change significantly with the change of the above reaction conditions. Thereupon, we synthesized a series of structurally well-defined glyco-CuInS_2_ QDs with natural carbohydrates on the surface.

### Structural characterization of glyco-CuInS_2_ QDs

**Fig. 1:**
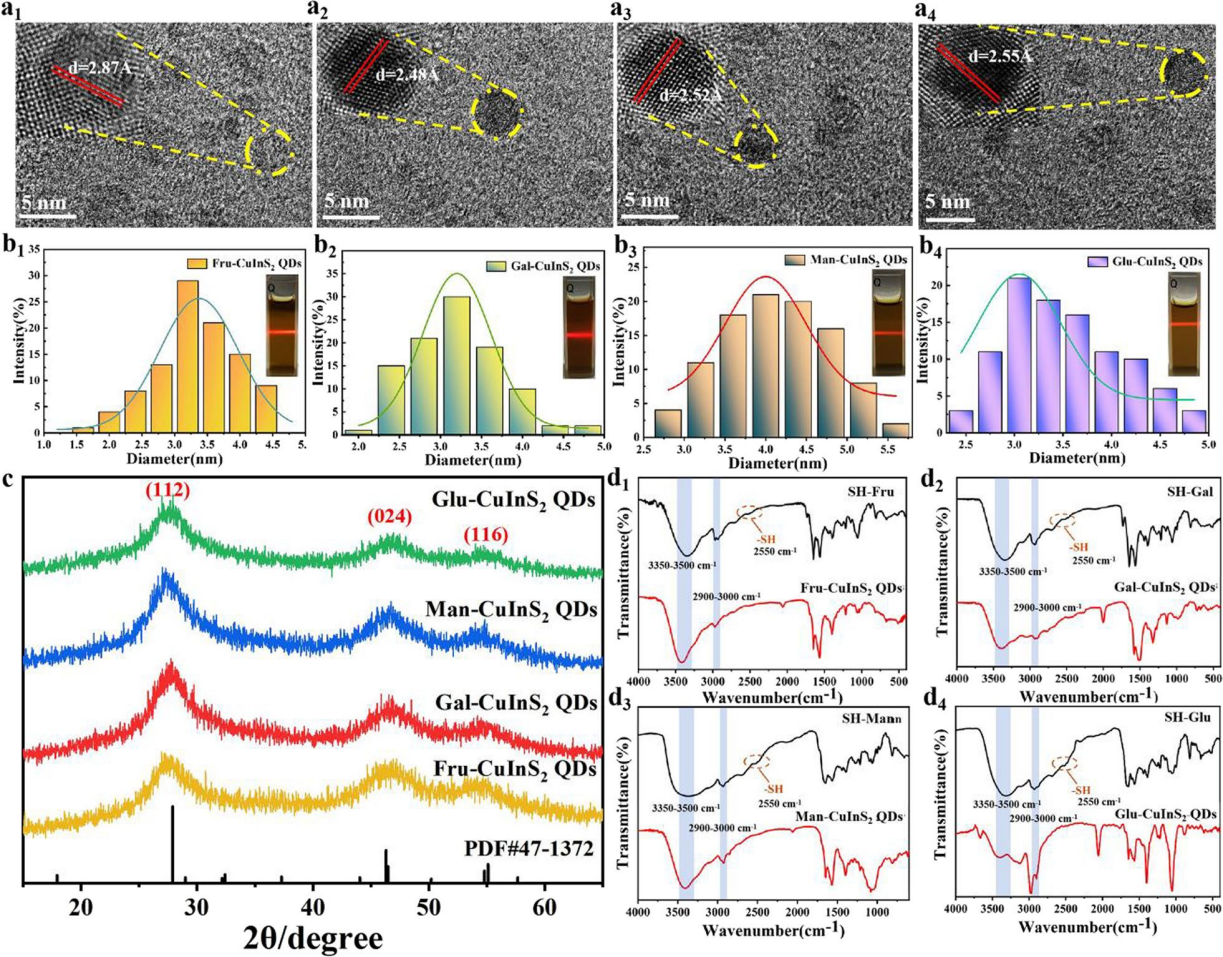
**a**_**1**_–**a**_**4**_ TEM images of Fru-CuInS_2_, Gal-CuInS_2_, Man-CuInS_2_ and Glu-CuInS_2_ QDs at a scale bar of 5 nm, respectively (Inset: HR-TEM images of glyco-CuInS_2_ QDs and their lattice spacing). **b**_**1**_–**b**_**4**_ Particle size distribution maps for Fru-CuInS_2_, Gal-CuInS_2_, Man-CuInS_2_ and Glu-CuInS_2_ QDs (Inset: Path diagram of glyco-CuInS_2_ QDs irradiated by laser pointer under daylight). **c**_**1**_ XRD diagram of Glu-CuInS_2_ QDs synthesized with four monosaccharides as ligands. **d**_**1**_–**d**_**4**_ FTIR spectra of Fru-CuInS_2_, Gal-CuInS_2_, Man-CuInS_2_ and Glu-CuInS_2_ QDs for each of four sulfhydrylated monosaccharides and their synthetic glyco-CuInS_2_ QDs

These synthesized glyco-CuInS_2_ QDs were characterized by TEM, DLS, XRD, FT-IR and XPS to demonstrate whether CuInS_2_ QDs were successfully encapsulated by the peripheral sulfhydryl monosaccharides, respectively. The effect of monosaccharide composition on crystal structure and morphology of CuInS_2_ QDs was first confirmed. As shown in the TEM images of glyco-CuInS_2_ QDs (Fig. 1 a_1_–a_4_), all monosaccharide-capped CuInS_2_ QDs have regular spherical shape and good monodispersity with mean diameters in the range of 3–4 nm in aqueous phase (Fig. 1b_1_–b_4_). Meanwhile, these insets to Fig. 1 a_1_–a_4_ show clear crystal lattice fringes with lattice spacing of 2.52 Å, 2.48 Å, 2.87 Å and 2.55 Å, respectively, which indicates the single crystal nature of the QDs. Figure 1c shows the XRD patterns of these synthesized glyco-CuInS_2_ QDs. The XRD spectra in Fig. 1c confirm the crystal structure of these glyco-CuInS_2_ QDs. The XRD pattern of glyco-CuInS_2_ QDs consisted of three major peaks with 2θ values of 27.9, 46.3, and 54.8, respectively, and all diffraction peaks corresponding to the (112), (024), and (116) indices of the tetragonal crystal structure (CuInS_2_, JCPDS no. 47-1372), respectively, suggesting that the crystal structure is well-maintained after capping with monosaccharides. According to the Debye–Scherrer formula: D = Kλ/Bcosθ (Where θ is the diffraction angle; λ is the X-ray wavelength; B is the half-height width of the diffraction peak; K = 0.89), the sizes of the prepared quantum dots were calculated to be 3.53 nm, 3.89 nm, 4.11 nm and 3.40 nm, respectively, which were consistent with the TEM and particle size test results. Furthermore, the clear lattice fringe (inset of Fig. 1a) indicated that the crystal structure of these CuInS_2_ QDs are not affected after capping with four monosaccharides. In addition, the broad diffraction peaks suggest that they have small sizes [28]. Besides, no other diffraction peaks were observed, which emphasizes the absence of any impurities in the prepared samples.

Moreover, the IR spectrum reveal the composition of as-synthesized glyco-CuInS_2_ QDs. The FTIR spectra of the sulfhydrylated monosaccharides and sugar-capped CuInS_2_ QDs are compared in Figure d_1_-d_4_. The IR absorption bands of four sulfhydrylated monosaccharides at 3500–3000 cm^−1^ are ascribed to the stretching vibration of -OH, whereas the peaks at 3000–2900 cm^−1^, 2500–2600 cm^−1^ and 1750 cm^−1^ are assigned to -CH_2_-, -SH and C = O of the ester groups, respectively. Because the CuInS_2_ QDs are capped with monosaccharides, typical peaks of sugars can be found in the IR spectrum of glyco-CuInS_2_ QDs. More importantly, the almost disappearance of the -SH stretching vibrational peak indicates that these sulfhydrylated monosaccharides may combine on the surface of the CuInS_2_ through the -SH groups [29]. SEM–EDS spectrum of glyco-CuInS_2_ QDs had been performed in Additional file 1: Fig. S6 to further determine the elemental composition of these QDs. These results indicate that all of glyco-CuInS_2_ QDs contain C, O, Cu, In, and S elements. Meanwhile, the distribution of elements of CuInS_2_ QDs are also studied with EDX elemental mappings, the bright points indicate the high concentration of the elements in Additional file 1: Fig. S7. The results show that C, O, Cu, In, and S elements are homogeneously distributed throughout sample, suggesting that CuInS_2_ QDs are successfully capped with four monosaccharides, respectively.

**Fig. 2:**
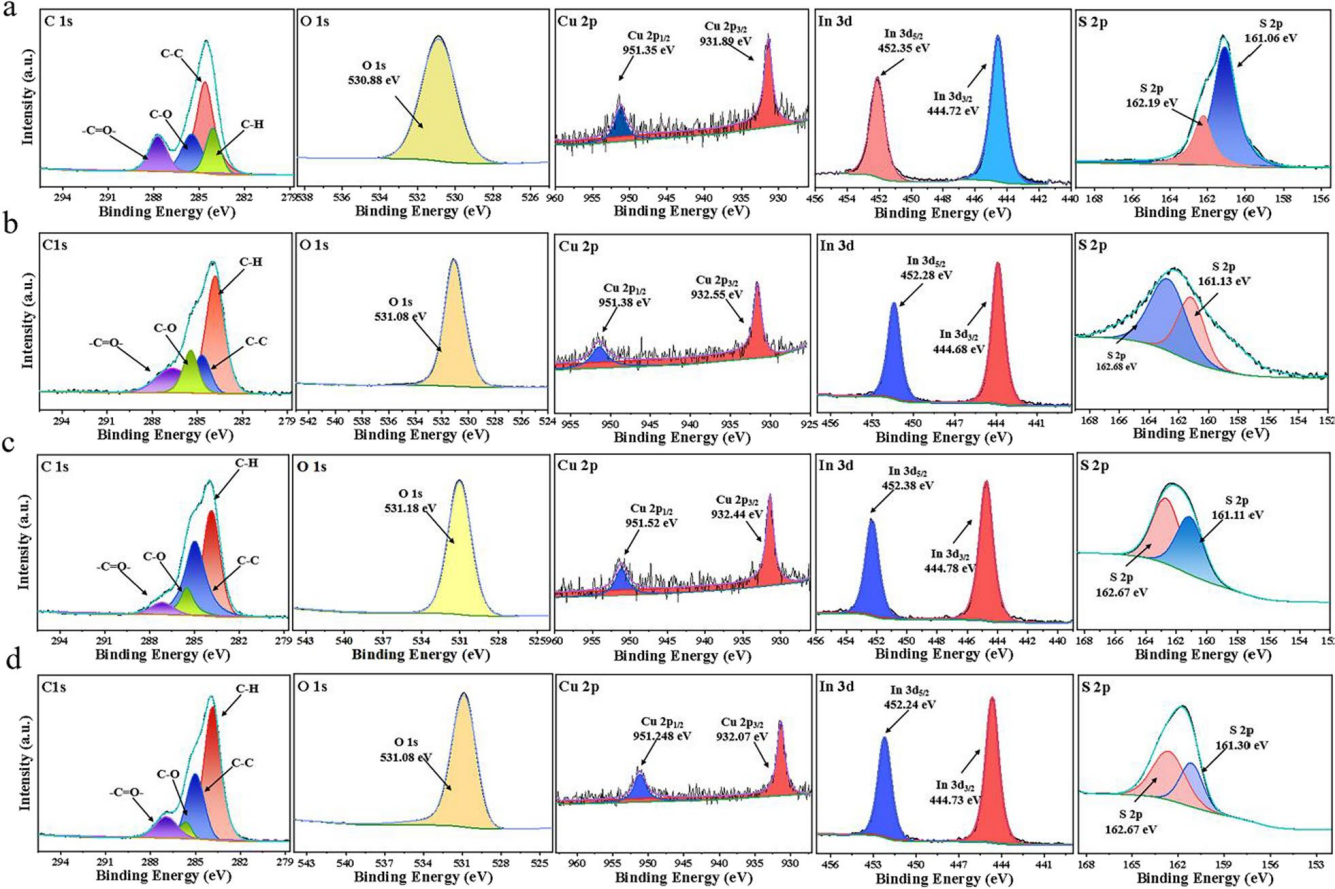
**a**–**d** High-resolution XPS spectra of C 1 s, O 1 s, Cu 2p, In 3d and S 2p of Fru-CuInS_2_, Gal-CuInS_2_, Man-CuInS_2_ and Glu-CuInS_2_ QDs, respectively

Besides, XPS was used to further confirm the composition and the valence state of all elements structure in these monosaccharide-capped CuInS_2_ QDs (Additional file 1: Fig. S8). The XPS survey scans for glyco-CuInS_2_ QDs clearly displayed the C 1 s, O 1 s, Cu 2p, In 3d, and S 2p signals at around 285, 531, 952, 452 and 163.3 eV, respectively [30, 31]. To obtain deeper information about CuInS_2_ QDs and structure, the high-resolution XPS spectra were collected and analyzed. The high-resolution scan of the C 1 s spectrum (Fig. 2) can be fitted with four peaks at 284.1 (C-H), 284.6 (C–C), 285.5 (C–O) and 287.8 (C = O) eV [32, 33], and these results are in accordance with the results obtained by FTIR spectrum. Meanwhile, the high-resolution O 1 s spectra show the signals centered at 531 eV originating from the carboxyl oxygen (C = O) [34]. The Cu 2p core level is divided into two peaks representing Cu 2p_3/2_ (~ 932 eV) and Cu 2p_1/2_ (~ 951 eV), confirming the valence state of ions is + 1 rather than + 2 due to the disappearance of the peak at 944.0 eV for Cu^2+^ (Fig. 2) [35]. Therefore, the results indicated that the oxidation state of Cu^2+^ was reduced by sulfhydryl monosaccharide in the process of CuInS_2_ QDs preparation. Moreover, the valences states of In^3+^ (In 3d_5/2_, ~ 445 eV and In 3d_3/2_, ~ 452 eV) is also confirmed by XPS spectra [30]. Finally, the S is identified as -2 by analyzing the XPS spectra of S2p [35]. Thus, the existence of monosaccharide on the surface of CuInS_2_ QDs can be proved.

**Fig. 3:**
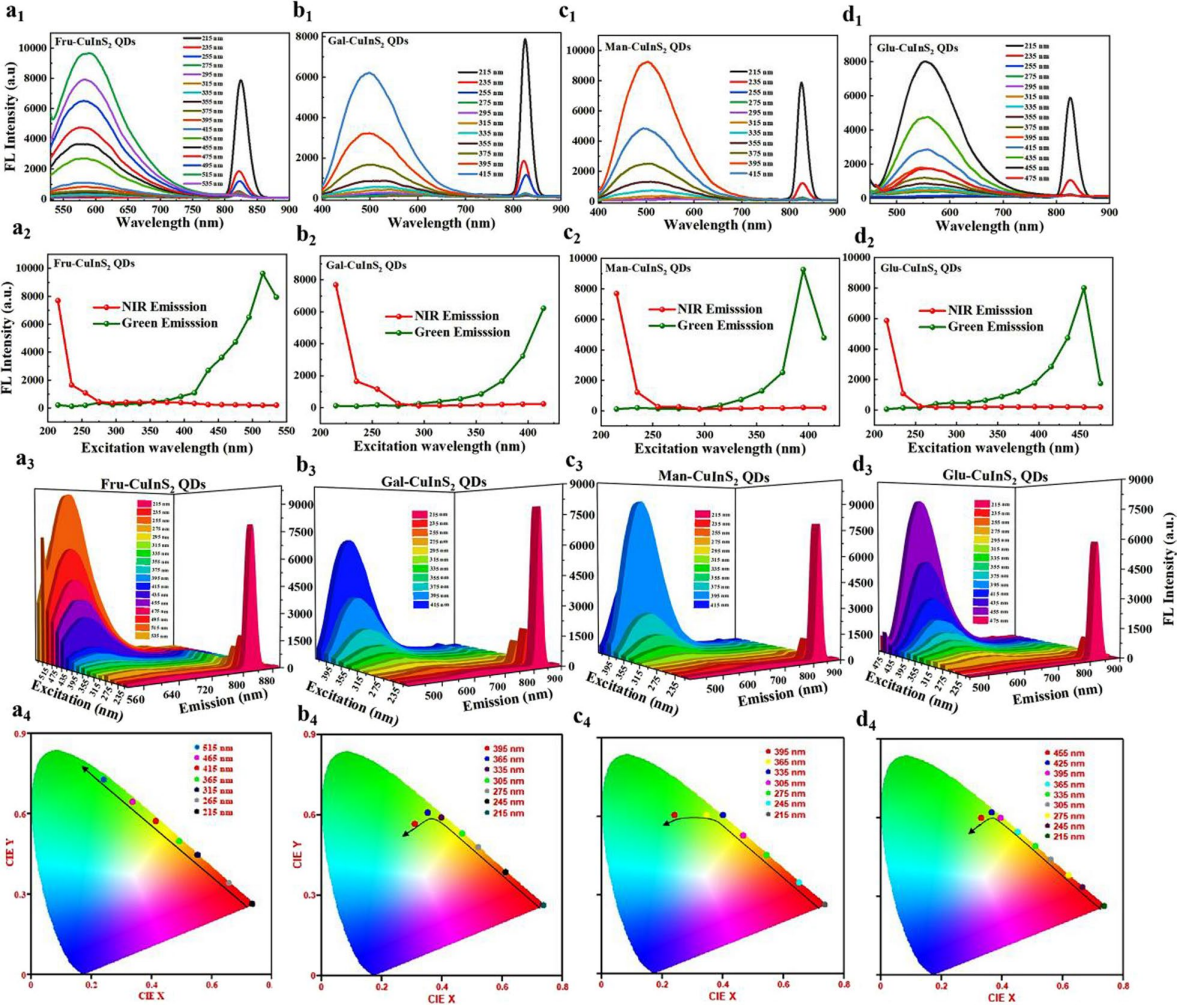
Computational models **a** CuS_2_ model, **b** CuInS_2_ (001) model, **c** Fructose model, and **d** Fructose adsorbed on CuInS_2_ (001) model. Geometric optimization models **e** Fructose model, **f** Fructose adsorbed on CuInS_2_ (001) model, (**g**) CuInS_2_ (001) charge hair distribution; red: O, yellow: S, orange: Cu, brown: In, gray: C, black and gray: fixed atoms (bond length in Å; bond angle in)

Furthermore, it is important to establish the long-term structure and dimensional stabilities of QDs materials. Firstly, the zeta potentials of the synthesized CuInS_2_ QDs were determined by means of a zeta potential analyzer. According to previous reports [36], solutions with a zeta potential above + 20 mV and below − 20 mV were considered stable. As can be seen in Additional file 1: Fig. S9, the zeta potentials of the four ligand-synthesized QDs were − 30.1 mV, − 28.7 mV, − 23.9 mV, and − 25.2 mV, respectively, indicating that these glyco-CuInS_2_ QDs are stable in solution. It is noteworthy that there is a slight variation in the zeta potential values of these QDs when the surface charge density increases, indicating a reduced tendency to aggregate due to electrostatic repulsion [37]. In addition, the chemical properties of the four CuInS_2_ QDs were stable, as all the glyco-CuInS_2_ QDs still exhibited regular spherical morphology with an average diameter of about 4 nm after day fourteen, with good dispersion, thus ensuring the dimensional stability of these synthesized CuInS_2_ QDs (Additional file 1: Fig. S10).

### Simulation study of Fru-CuInS_2_ QDs as an example

#### Calculation method

In this study, the DMol_3_ package in Material Studio was used to complete the calculations using a first-principles pseudopotential plane wave algorithm based on Koho-Sham self-consistent density generalization theory [38, 39]. The exchange–correlation energy was calculated using the generalized gradient approximation (GGA) and the Perdew-Burke-Ernzerhof (PBE) pseudopotential [40], according to which the interaction between the fructose adsorbed valence electrons and ions in the CuInS_2_ system can be described approximately. The cut-off energy is set at 489.8 eV for the optimization of the system structure, energy, density of states and energy band correlation calculations. The Brillouin zone integral uses a symmetric k-point method in Monkhors-Pack form, dividing the lattice according to 1 × 1 × 1. To avoid the interaction of repeated cycles of the surface in the z-axis direction, a vacuum layer of 10 Å is constructed above the surface. The convergence criteria for the electron self-consistent interaction energy, the force on all atoms and the maximum atomic displacement are 2 × 10^–5^ Ha, 0.004 Ha/Å and 0.005 Å respectively. These data were chosen as optimal values based on convergence verification.

#### Computational models

The tetragonal CuS_2_ with formation energy, density and band gap of -0.540 eV, 4.22 g/m^3^ and 0 eV were selected as 3.650 Å, 4.723 Å and 5.826 Å for a, b and c, as shown in Fig. 3a. The CuS_2_ doped in system with CuS_2_ (001) surface not only has a low surface energy but also is more easily exposed under typical adsorption reaction conditions. When Cu/In is 1/1, In provides the active site for fructose, which exhibits excellent catalytic performance as shown in Fig. 3b. Fructose is shown in Fig. 3c. In this study p (4 × 4) with 56 atoms were constructed using CuS_2_ as the substrate, with 12 In atoms doped on the surface of CuS_2_ (001) and cell sizes a, b and c of 11.599 Å, 11.599 Å and 15.799 Å, as shown in Fig. 3d.

**Fig. 4:**
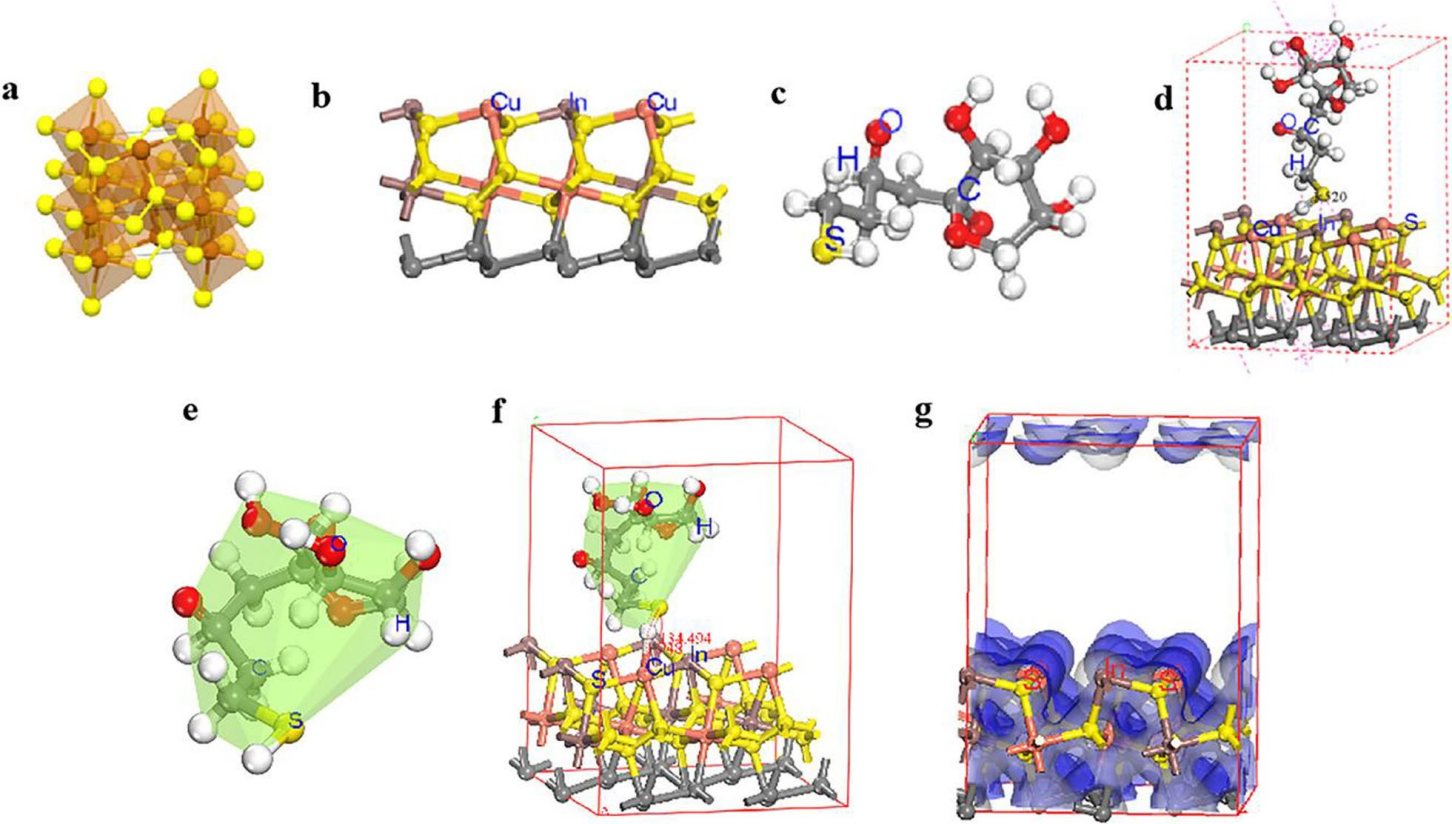
**a**_**1**_–**d**_**1**_ Dual fluorescence spectra of glyco-CuInS_2_ QDs at different excitation wavelengths; **a**_**2–**_**d**_**2**_ Dual fluorescence trend maps of glyco-CuInS_2_ QDs at different excitation wavelengths; **a**_**3**_–**d**_**3**_ Fluorescence 3D images of glyco-CuInS_2_ QDs at different excitation wavelengths; **a**_**4**_–**d**_**4**_ CIE chromaticity maps of glyco-CuInS_2_ QDs at different excitations

In the adsorption energy calculation, the atoms in the lowermost atomic layer are held in their initial positions in the equilibrium structure, while the top two atomic layers and the adsorbate are both released, and the system is geometrically optimized to reach a steady state [41], the adsorption energy (E_ads_) of Fructose adsorbed on the CuInS_2_ (001) surface is defined as:

$$E_{{{\text{ads}}}} = E_{{({\text{TG}})}} + E_{{({\text{Fru}})}} - E_{{({\text{Total}})}}$$

where: E_(TG)_ is the total energy of the in doped CuS_2_ (001) system; E_(Fru)_ is the energy of a single fructose molecule; E_(Total)_ is the total energy of a fructose molecule adsorbed on the CuInS_2_ (001) system.

#### Calculation results and discussion

Firstly, the fructose molecular structure was optimized to obtain the most stable structure as shown in Fig. 3e. For the CuS_2_(001)-doped in system, the geometric optimization was performed to obtain the most stable optimization was performed to obtain the most stable condition of the system as shown in Fig. 3b. Then the adsorption of fructose molecules on the CuInS_2_(001) surface was considered, and the adsorption configuration was represented by Fru-CuInS_2_ as shown in Fig. 3f. The charge distribution map of the CuS_2_(001)-doped in system was calculated by optimization, as shown in Fig. 3g. As shown in Fig. 3, the optimized fructose molecules adsorbed on the surface of CuInS_2_(001) were obtained through simulations, and a significant change in the S–H bond length was found, with the bond length changing from 1.520 Å to 1.918 Å. This indicates that the fructose molecules adsorbed on the surface of CuInS_2_(001) were activated, resulting in chemisorption. Furthermore, the results of calculation of the adsorption energy of fructose adsorption on CuInS_2_ are shown in Table 1. The fructose adsorption in the CuInS_2_(001) system shows excellent adsorption performance with an energy of 3.05 Ha. The corresponding DOS and PDOS calculations for this system are shown in Additional file 1: Fig. S11. A comparison of the DOS plots of the two systems shows a sharp peak at -4.2 eV and a small peak at the Fermi energy level, indicating that the fructose molecules are adsorbed on the surface of CuInS_2_(001). A comparison of the PDOS plots of the two systems shows a new energy band at -23 eV-20 eV and a small peak at the Fermi energy level, indicating that the fructose molecules are adsorbed on the surface of CuInS_2_(001) with good adsorption properties.

**Table 1:** Calculation of the adsorption energy of fructose adsorption on CuInS_2_

System model	E_k_/Ha	E_cor_/Ha	E_elc_/Ha	E_TG_/Ha	E_OH_^−^/Ha	E_total_/Ha	E_SA_/Ha	E_ads_/Ha
Fru-CuInS_2_	− 90.30034	4.919	68.103	− 101328.9702	− 1201.829586	− 102533.8534	− 102519.5038	3.0536553

*E*_*k*_ kinetic energy, *E*_*cor*_ exchange–correlation energy, *E*_*elc*_ electrostatic energy, *E*_*TG*_ total energy of doped CuS_2_ (001) system, *E*_*OH*_*-* the energy of a single OH^−^, *E*_*total*_ the total energy of the fructose absorbed system, *E*_*SA*_ sum of atomic energies, *E*_*ads*_ absorption energy

### Color-tunable emission of glycol-CuInS_2_ QDs

In order to understand the optical properties of these glyco-CuInS_2_ QDs and to open up a wider range of applications for these materials, their photophysical behavior has been investigated in depth. Firstly, UV–Vis absorption spectra of the as-prepared CuInS_2_ QDs are recorded in aqueous solutions (Additional file 1: Fig. S12). All of the glyco-CuInS_2_ QDs show strong absorption at 200–350 nm, assigned to the absorption bands of the thiolated monosaccharides. Besides, these UV–vis absorption spectra also show broad absorption from 400 to 800 nm without distinct excitonic absorption features, which is consistent with previous reports for QDs [42]. Therefore, the glyco-CuInS_2_ QDs exhibit wide absorption band covering the UV and Vis regions. Meanwhile, considerable absorption coefficient of the visible region is essential to exciting red or NIR fluorescence bioimaging in a confocal laser scanning microscopy (CLSM) system.

Subsequently, the photoluminescence (PL) behavior of four kinds of glyco-CuInS_2_ QDs were investigated at room temperature in detail. The emission spectra of glyco-CuInS_2_ QDs in aqueous solution with progressively longer excitation wavelengths from 215 to 535 nm in 20 nm increment are shown in Fig. 4a_1_–d_1_, respectively. It can be observed that all glyco-CuInS_2_ QDs exhibit two well-separated photoluminescence peaks in the visible light region (500 ~ 600 nm) and the NIR range (~ 827 nm) upon excitation spectral scan (215 nm to 515 nm), corresponding to the characteristic photoluminescence peaks of water-soluble CuInS_2_ QDs [43]. Recently, Arshad et al. also have reported an aqueous synthesis of CuInS_2_ QDs by using GSH as capping ligand, which showed two well-defined photoluminescence peaks at 550 nm and 725 nm [44]. The dual-emission phenomenon has a wide range of applications as chemosensors and markers for bioimaging [31, 45, 46]. Besides, glyco-CuInS_2_ QDs mainly emit NIR fluorescence at excitation wavelengths in the range 215–250 nm and green fluorescence in the range of 400–500 nm. As shown in Fig. 4a_2_-d_2_, these glyco-CuInS_2_ QDs show different green fluorescence emission maxima at 582 nm (Fru-CuInS_2_ QDs), 500 nm (Gal-CuInS_2_ QDs), 502 nm (Man-CuInS_2_ QDs) and 553 nm (Glu-CuInS_2_ QDs) under 515, 415, 395 and 455 nm excitation, respectively. And, all of glyco-CuInS_2_ QDs exhibit the same NIR fluorescence emission maxima at 827 nm under 215 nm excitation. The fluorescence quantum yields (QY) and lifetimes (τ) of these QDs for the green fluorescence emission in water were measured, respectively. As shown in Additional file 1: Table S2, the QYs of Fru-CuInS_2_, Gal-CuInS_2_ and Glu-CuInS_2_ QDs exceed 10% in water and Fru-CuInS_2_ QDs have highest QY (Φ_f_ = 15.05) compared with three other QDs (Φ_f,Gal-CuInS2_ = 10.05%, Φ_f,Man-CuInS2_ = 4.60%, Φ_f,Glu-CuInS2_ = 12.10%). Furthermore, the average fluorescence lifetimes for the maximum emission of glyco-CuInS_2_ QDs in water are 4.27 ns, 4.71 ns, 3.96 ns and 6.61 ns, respectively (Additional file 1: Table S2 and Fig. S13). Unfortunately, the QYs and lifetimes of QDs for the NIR emission at 827 nm cannot be obtained due to the lack of suitable excitation source (~ 215 nm) in instrument testing.

Notably, the green and NIR emissions are well separated by > 235 nm, which is extremely beneficial for biosensing or bioimaging. Surprisingly, these glyco-CuInS_2_ QDs have very large Stokes shift of up to 612 nm for NIR emission (Additional file 1: Fig. S14), which allows for efficient separation of absorbance and emission maxima. More interestingly, the fluorescence emissions of glyco-CuInS_2_ QDs can be tuned from green to NIR by switching the excitation wavelength. As shown in Fig. 4a_3_-d_3_, lower-energy emission peak at 827 nm is much higher than the higher-energy emission peak around 500 nm under 215 nm excitation. And then, the green light intensity gradually increases and the NIR light intensity decreases with the increase in the excitation wavelength from 215 to 515 nm. Ultimately, the green fluorescence is in a dominant position. In addition, the approximate luminescence color changes of glyco-CuInS_2_ QDs were identified with the help of chromaticity color coordinates in the CIE chromaticity diagram (Fig. a_4_–d_4_). The CIE chromaticity diagram results indicate that different light colors4 are produced on the standard swatches at different excitation wavelengths in the range of 215 to 515 nm, while the light colors are excessive from red to green. Therefore, the luminescence colors of these glyco-CuInS_2_ QDs could be easily adjusted through changing the excitation wavelength.

**Fig. 5:**
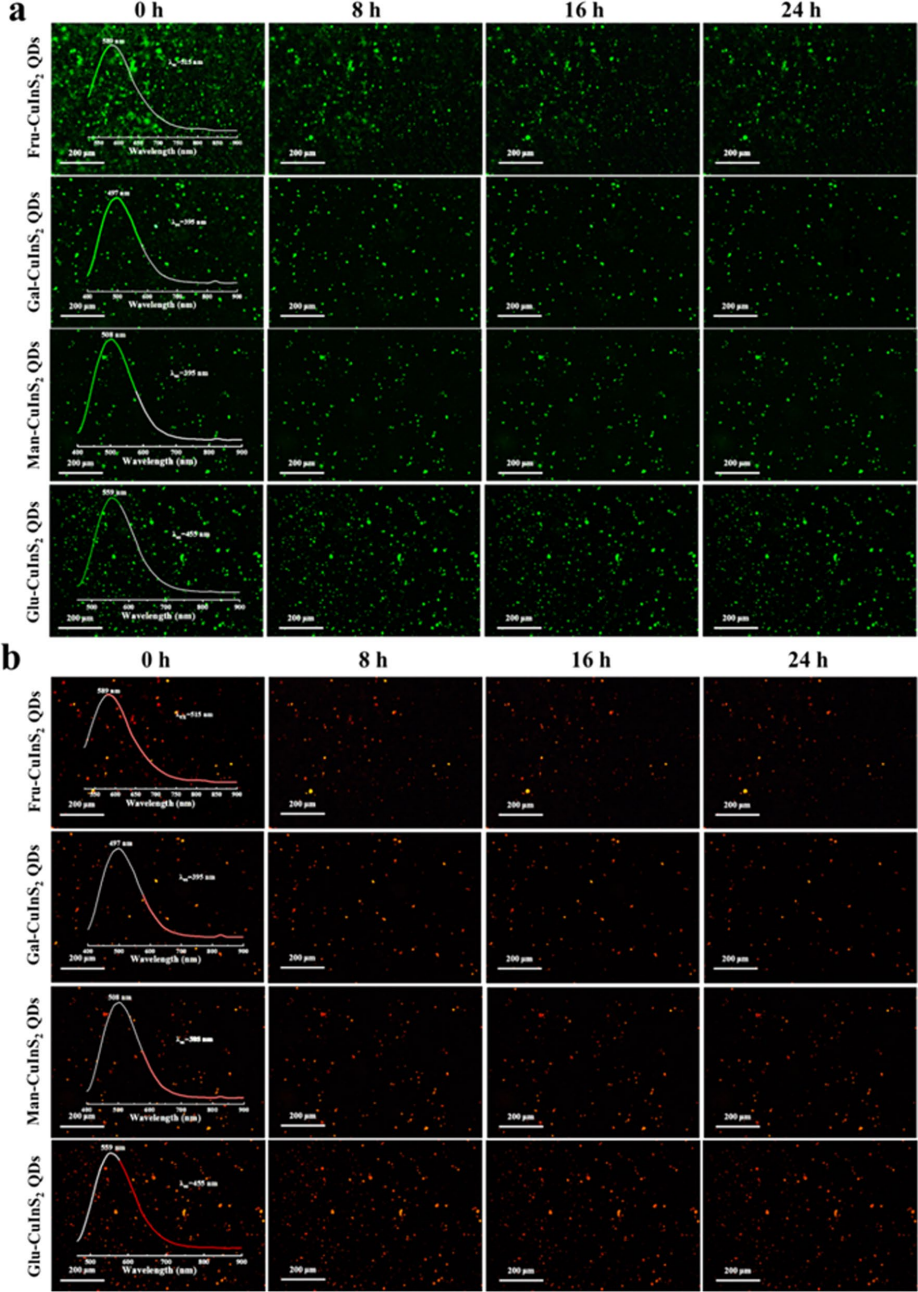
Fluorescence microscopy images of glyco-CuInS_2_ QDs, obtained from green **a** and red **b** emission channels continuously irradiated for 0 h, 8 h, 16 h, 24 h respectively (the images also include the emission spectra of QDs at different excitation wavelengths)

Moreover, the photostability of glyco-CuInS_2_ QDs is extremely important feature used as fluorescence probes in biological staining and cell imaging. Therefore, the luminescent stabilities of four glyco-CuInS_2_ QDs have been investigated using fluorescence spectroscopy. As shown in Additional file 1: Fig. S15, all glyco-CuInS_2_ QDs in aqueous solution show good photostability because there are no obvious changes between the fluorescence spectra of glyco-CuInS_2_ QDs at the first day, the seventh days and the fourteenth days. Furthermore, light illumination stabilities of glyco-CuInS_2_ QDs in the aggregated state are also investigated under the illumination of two light sources and times. Firstly, all these solid-state glyco-CuInS_2_ QDs were prepared by adding dropwise the concentrated solutions on the glass slides for drying. Then, these glass slides were viewed under a fluorescence microscope. Significantly, all these solid-state QDs exhibited strong fluorescence signals irradiated by altering the excitation wavelengths in both of the green and red channels (Fig. 5). Besides, the luminescent brightness did not decrease significantly after 24 h of illumination. These test results also identify that all glyco-CuInS_2_ QDs have excellent light-stabilities.

### Formation and dual emission mechanisms of glyco-CuInS_2_ QDs

**Fig. 6:**
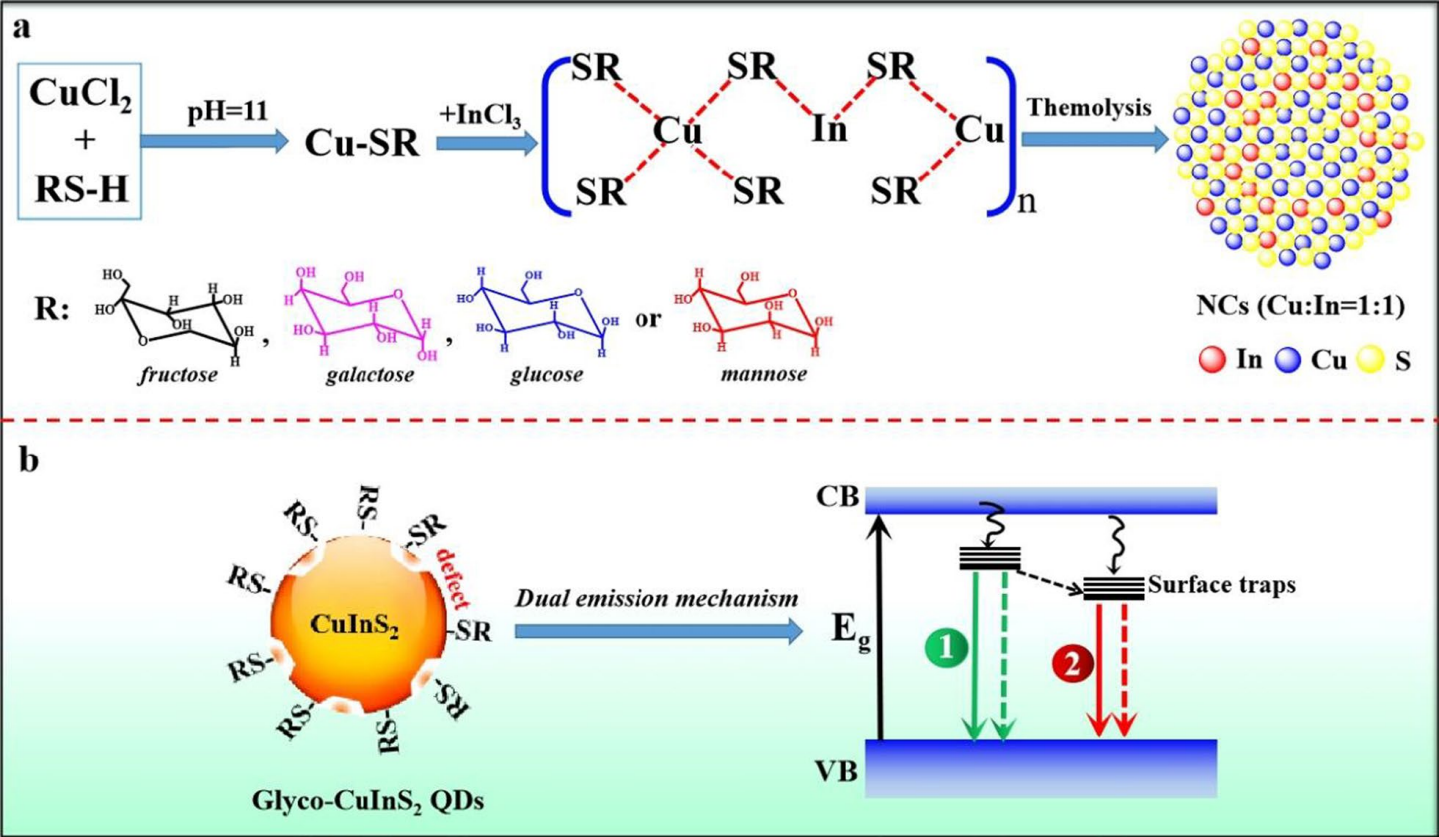
**a** Schematic illustration of formation mechanism of glyco-CuInS_2_ QDs; **b** Schematic diagram of semiconductor QDs photoluminescence (Solid lines represent radiative transitions, dashed lines represent non-radiative transitions)

The formation mechanism was summarized in Fig. 6. As shown in Fig. 6a, Cu^+^ and In^3+^ cations were released from metal precursors and coordinated with thiol-ending glycosides to form Cu-In thiolate [CuIn(SR)_x_]_n_ after being mixed with thiol-ending monosaccharides (RS-H). Subsequently, [CuIn(SR)_x_]_n_ was decomposed to QDs by thermolysis process. The formation of glyco-CuInS_2_ QDs could be explained by a nucleation-growth mechanism following the LaMer model [47]. Meanwhile, based on the hard-soft acid–base (HSAB) theory, Cl^−^ is hard base, while Cu^+^ and In^3+^ are soft and hard acid, respectively. So, Cu^+^ is much easier to be released from CuCl than In^3+^ ions from InCl_3_ due to the weaker interaction between Cu-Cl. On the other hand, RSH is soft base. So, the Cu-rich [CuIn (SR)_x_] _n_ is formed because of the stronger coordinating ability between Cu^+^ and RS^−^ than that between In^3+^ and RS^−^ [48]. Thus, highly Cu-rich glyco-CuInS_2_ QDs are finally obtained with high density of surface defect states.

Dual emission mechanism of glyco-CuInS_2_ QDs can further be elucidated based on their absorption and emission behavior. As shown in Fig. 6b, the energy band structure usually consists of a low-energy valence band (VB) filled with electrons and an empty high-energy conduction band (CB). The photon energy is transferred to the QDs if they can absorb incident light under laser excitation. Thus, under the action of high-energy rays, the electron jumps from the fundamental energy level of the valence band to the higher energy level of the conduction band. Subsequently, the photogenerated electrons are transferred nonradiatively to the intra-bandgap states (1). Meanwhile, the surface defects on the QDs capture some electrons, resulting in the intra-bandgap states (2). All electrons from two states undergo radiative and nonradiative recombination with holes [49]. Therefore, the emission monitored in the visible light region of 500 ~ 600 nm (high energy band) and the emission recorded at 827 nm (low energy band) may be attributable to visible excitonic emission and near-infrared surface defect emission, respectively. Indeed, this dual emission mechanism is consistent with previous published works on the two distinct transitions in Cu_x_InS_2_ QDs [50, 51].

### The pH-sensitive qualities of fluorescence

Due to the aggressive proliferation of tumor cells and the rapid formation of irregular blood vessels, nutrients and oxygen are rapidly consumed at the tumor site, causing lactic acid metabolites produced by glycolysis in the tumor cells to accumulate in the tumor interstitium, ultimately leading to a drop in the pH of the extra-tumor cellular environment to 6.2–6.9 [52]. Therefore, the high brightness of fluorescence probes in a weak acid solution is of great importance for in vivo imaging. Thus, it is essential to examine the effects of pH on the fluorescence intensity of glyco-CuInS_2_ QDs. As shown in Additional file 1: Fig. S16, the fluorescence of four glyco-CuInS_2_ QDs exhibit pH-sensitive response, and the maximum fluorescence intensity can be obtained when the pH is in the range of 6 to 7, which will be beneficial to the in vivo imaging.

### The dual-color live-cell membrane imaging of glycol-CuInS_2_ QDs

Motivated by these excellent optical performances of glycol-CuInS_2_ QDs, we further study their imaging performances in living organisms. Three tumor cells (HeLa, A549, MKN-45) were selected for testing prior to performing cell imaging experiments. Firstly, the detection of cytotoxicity is a critical prerequisite for its application in cell imaging. Three kinds of tumor cells were incubated in concentrations of 20, 40, 60, 80 and 100 μg/mL of Fru-CuInS_2_, Gal-CuInS_2_, Man-CuInS_2_ and Glu-CuInS_2_ QDs aqueous solutions for 24 h, and their effects on cell viability were determined by MTT [53, 54], respectively. As shown in Additional file 1: Fig. S17, the survival rates of three tumor cells are above 90% for all culture concentrations, which indicated that all glycol-CuInS_2_ QDs possesses low cytotoxicity towards the living cells.

**Fig. 7:**
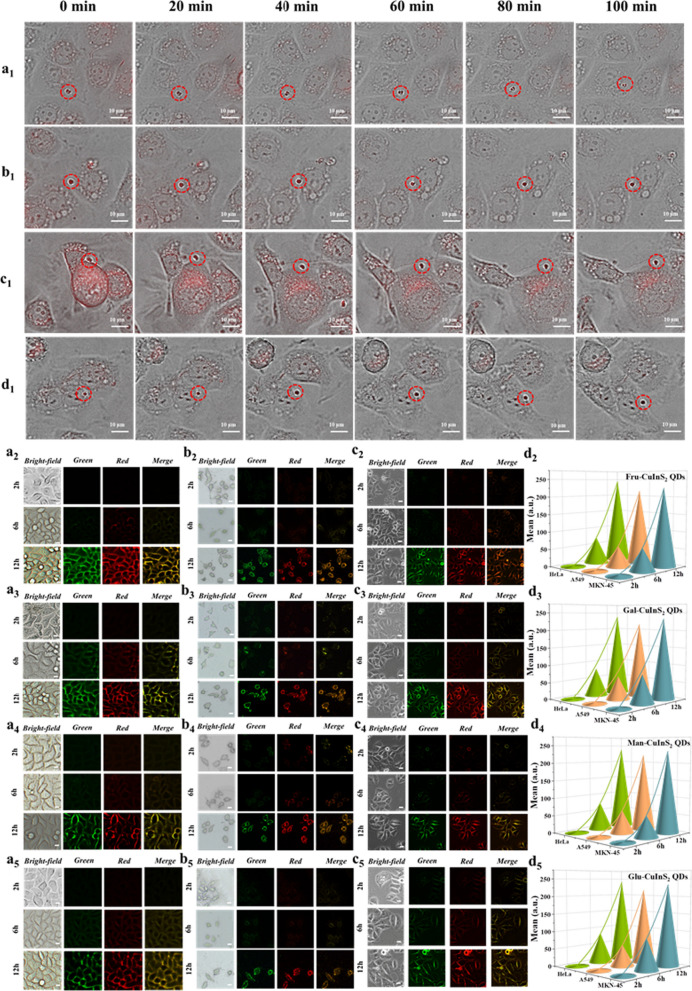
**a**_**1**_–**d**_**1**_ the images of 0–100 min migration of aggregated glyco-CuInS_2_ QDs towards the cell membrane, respectively; **a**_**2**_–**a**_**5**_ the imaging images of HeLa cells treated with 100 μg/mL glyco-CuInS_2_ QDs for 2 h, 6 h and 12 h, respectively; **b**_**2**_–**b**_**5**_ the images of A549 cells treated with 100 μg/mL glyco-CuInS_2_ QDs for 2 h, 6 h and 12 h, respectively; **c**_**2**_–**c**_**5**_ the imaging images of MKN-45 cells treated with 100 μg/mL glyco-CuInS_2_ QDs for 2 h, 6 h and 12 h, respectively (scale bar 10 μm); **d**_**2**_–**d**_**5**_ mean brightness values of HeLa, A549, MKN-45 cells after incubation with glyco-CuInS_2_ QDs for 2 h, 6 h and 12 h

Besides, their abilities to enter tumor cells are also essential for biological imaging. Therefore, we investigated how glycol-CuInS_2_ QDs entered three kinds of tumor cells before performing cell staining. The dynamical interaction process of glycol-CuInS_2_ QDs with tumor cells at different time points through a live cell workstation (Olympus DP80) were recorded in Fig. 7a_1_–d_1_, respectively. It can be observed that the aggregated QDs particles slowly enter the cell membrane with time migration from 0 to 100 min. These results indicate that all glycol-CuInS_2_ QDs enter target cells by endocytosis, which occurs at the cell surface. The low cytotoxicities and excellent cell-entry abilities of these glycol-CuInS_2_ QDs provide strong in vivo evidences for live cell staining.

Subsequently, cell imaging experiments were performed to further demonstrate the practical application of four glycol-CuInS_2_ QDs. The HeLa, A549, MKN-45 cells were individually isolated in 12-well plates and incubated with glycol-CuInS_2_ QDs at a concentration of 100 μg/mL for 2, 6 and 12 h, respectively. As shown in Fig. 7, all glycol-CuInS_2_ QDs are highly concentrated in HeLa, A549, MKN-45 cell membrane and the fluorescence signals are continuously enhanced with the incubation time increased. For example, almost no fluorescent signals are observed in membrane of three tumor cells because only a few Fru-CuInS_2_ QDs enter cells after 2 h in Fig. 7a_2_–c_2_. With the entry of more QDs in cell membrane after 6 h incubation, the increasing fluorescent signals can be observed. Finally, significant fluorescent signals are observed after 12 h incubation, suggesting that Fru-CuInS_2_ QDs can be effectively captured by cell membrane. Meanwhile, Gal-CuInS_2_ QDs, Man-CuInS_2_ QDs and Glu-CuInS_2_ QDs also exhibit similar imaging abilities, respectively (Fig. 7a_3_–c_3_, Fig. 7a_4_–c_4_ and Fig. 7a_5_–c_5_). Furthermore, all glycol-CuInS_2_ QDs show same dual-colorful and tunable fluorescence signals by altering the excitation wavelengths in green and red emission channels.

Besides, methods of imaging provide the means to visualize in space and time, but there is still a great need to make quantitative measurements. Image J is an open-source image processing platform for multidimensional image data, built for the specific needs of scientific images [55]. In this paper, we made use of the Image J software toolkit, which has utility in scientific image analysis, to quantify the imaging fraction. For example, as shown in Fig. 7d_2_, the specific fluorescence intensities of HeLa, A549 and MKN-45 cells incubated with Fru-CuInS_2_ QDs are 1.450 a.u, 3.069 a.u. and 5.748 a.u after 2 h of incubation, and increase to 75.656 a.u., 59.634 a.u. and 64.705 a.u. after 6 h of incubation, and significantly increase to 243.413 a.u., 214.783 a.u. and 225.490 a.u. after 12 h of incubation, respectively. Meanwhile, the specific fluorescence intensities of HeLa, A549 and MKN-45 cells incubated respectively with Gal-CuInS_2_ QDs, Man-CuInS_2_ QDs and Glu-CuInS_2_ QDs also exhibit the similar growth trends with the increasing of incubation time (Fig. 7d_3_–d_5_).

**Fig. 8:**
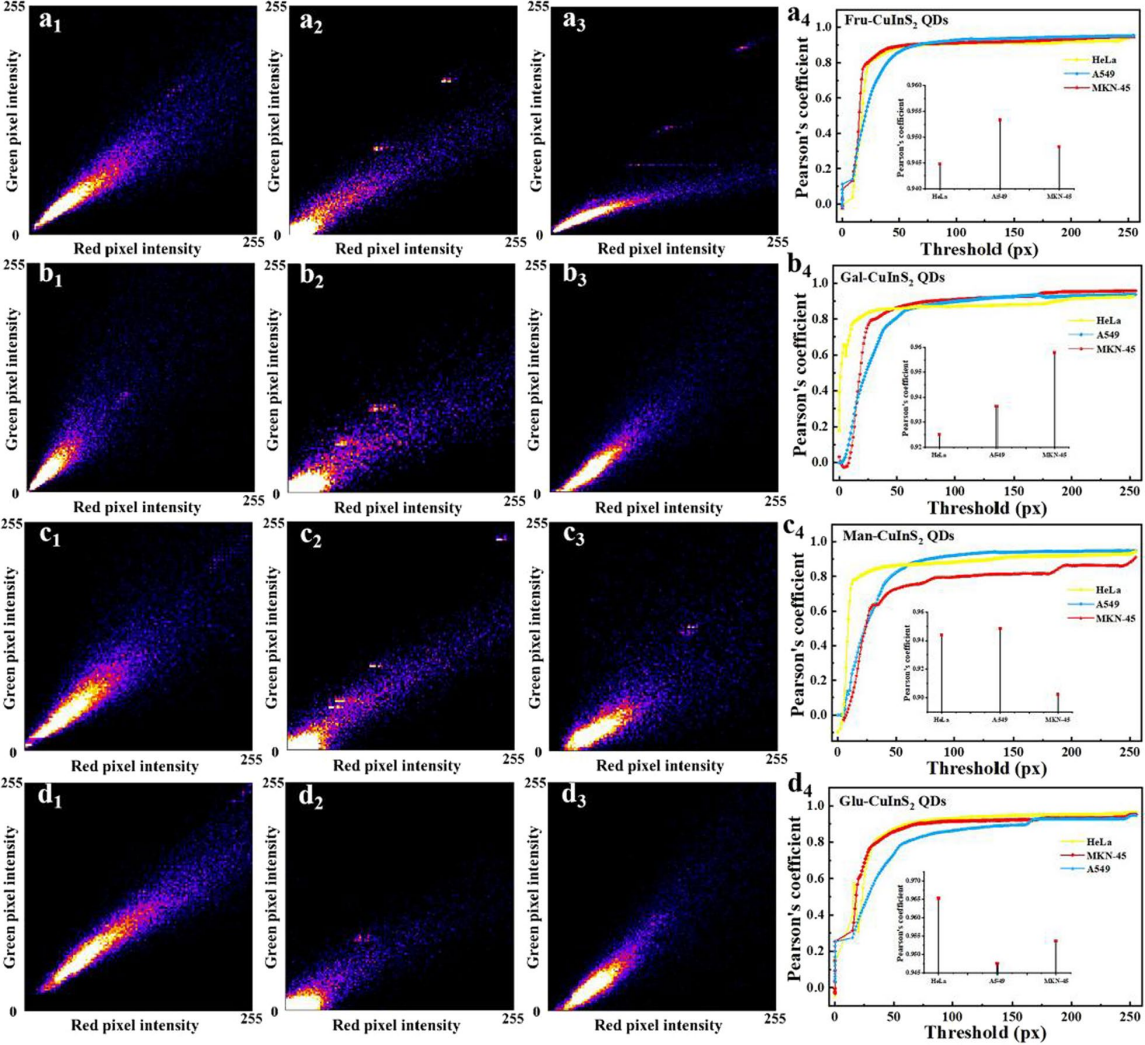
**a**_**1**_–**d**_**1**_ Pixel plots of imaging confocal after treatment of HeLa cells with Fru-CuInS_2_, Gal-CuInS_2_, Man-CuInS_2_ and Glu-CuInS_2_ QDs, respectively; **a**_**2**_–**d**_**2**_ Pixel plots of imaging confocal after treatment of A549 cells with Fru-CuInS_2_, Gal-CuInS_2_, Man-CuInS_2_ and Glu-CuInS_2_ QDs, respectively; **a**_**3**_–**d**_**3**_ Pixel plots of imaging confocal after treatment of MKN-45 cells with Fru-CuInS_2_, Gal-CuInS_2_, Man-CuInS_2_ and Glu-CuInS_2_ QDs, respectively; **a**_**4**_–**d**_**4**_ Plots of threshold versus Pearson correlation coefficient in fluorescence confocal detection (inset: overlap of different channel imaging positions for the three cells)

Most notably, confocal imaging experiments show the excellent specific cytomembrane location for living tumor cells. According to a previously reported literature [56], based on the recognition of glyco-cell membrane receptor proteins, nanodots with more glycosyl modifications are more recognized by the receptor, entered the cells more and fluoresced brighter. Thus, these glycol-CuInS_2_ QDs can specifically identify cancer cells with glyco-receptor protein overexpression on the cell membrane for fluorescence imaging. The cell membrane is involved in various cellular processes and biological functions, such as cell signalling, cell adhesion, endocytosis, cytosol and selective permeation of substances, therefore, the cell membrane is indispensable to the cell, and its observation can also yield much information related to cellular state and disease. In view of this, the synthesis of cell membrane-targeted fluorescent bioprobes is of great importance [57]. Furthermore, in order to determine whether the fluorescence emitted by the two channels remains in the same position, a fluorescence co-localization assay was performed [58]. From the scattering trend of pixel points in the co-localized pixel plots in Fig. 8, we can find that the fluorescence of glycol-CuInS_2_ QDs emitted by the red and green channels are in a somewhat linear relationship and favor the red channel, indicating that the red and green fluorescence are co-localized in a certain proportion, but the green fluorescence is slightly darker than the red fluorescence. We then carried out co-localization analysis by a series of different parameters, Pearson's correlation coefficient (PCC) [59]. Figure 8a_4_–d_4_ shows the overlap rates of these glycol-CuInS_2_ QDs in different cell imaging with red and green channel imaging. Reassuringly, all overlaps are above 90%, indicating high targeting specificity.

### The imaging of glycol-CuInS_2_ QDs in multicellular tumor spheroids

**Fig. 9:**
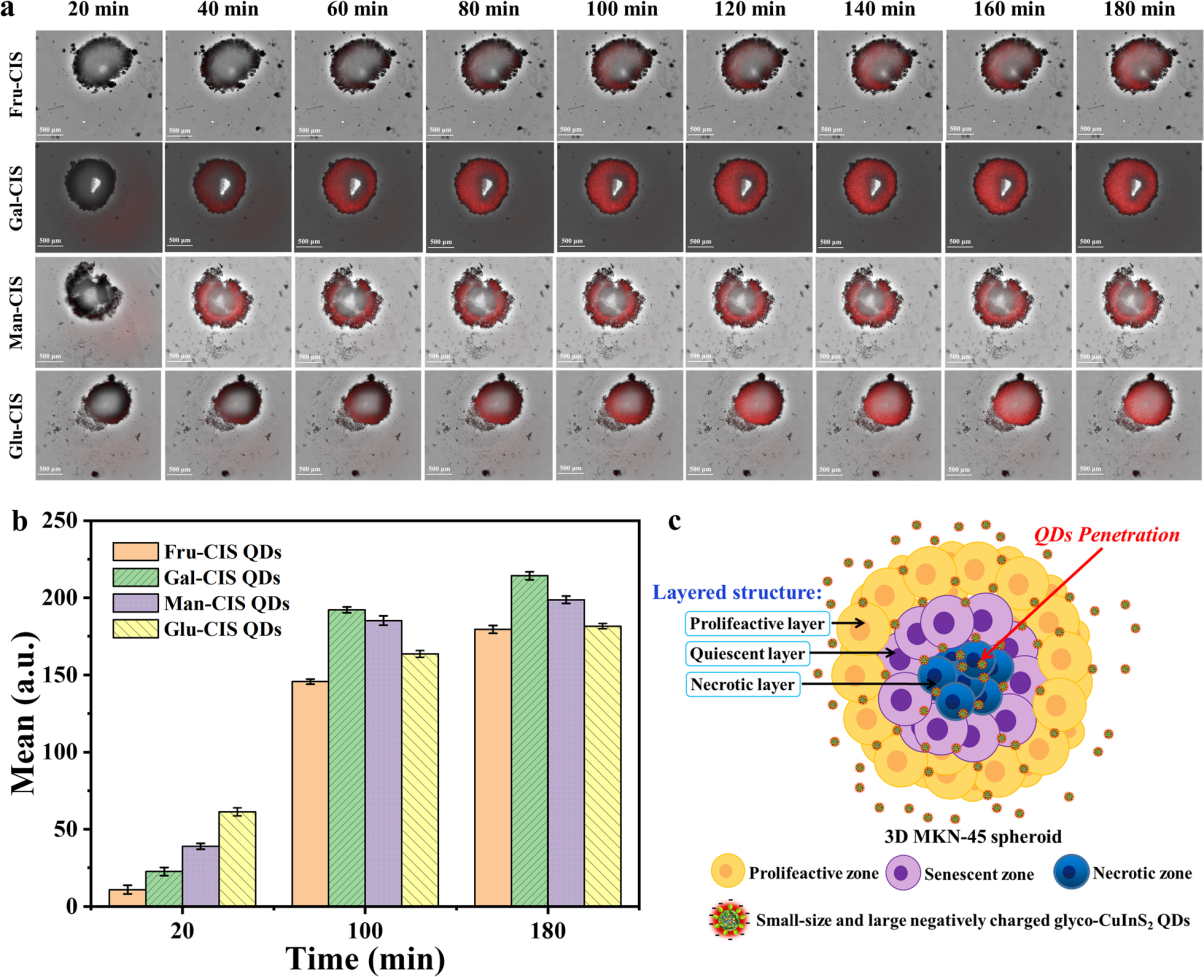
**a** Fluorescence expression of glyco-CuInS_2_ QDs in 3D MKN-45 cell microspheres at different times (Scale bar: 500 µm); **b** the corresponding quantitative fluorescence intensity values; **c** Schematic representation of glyco-CuInS_2_ QDs penetration in 3D MKN-45 spheroid

The studies of QDs imaging had been carried out in vitro with cancerous cell lines. However, only research in 2D monolayers model is unable to evaluate some very important abilities for glycol-CuInS_2_ QDs in cancer imaging, for example the ability of QDs to penetrate and retain within a tumor. At present, the challenge associated with QDs is their poor penetration depth, constraining them to a peripheral accumulation in in vivo tumors. Multicellular tumor spheroids (MCTS) have gained increased recognition as a useful three-dimensional (3D) tumor spheroid model for evaluating the effect of exogenous molecules on tumor progression in cancer research because they may closely mimic some physiological characteristics of solid tumors. However, to date, only few studies were conducted to investigate the QDs imaging in 3D in vitro spheroids models. Therefore, MCTS are used to evaluate the tumor penetration capacity of four glycol-CuInS_2_ QDs in this study, respectively (Additional file 1: Video S1–S4). Figure 9a shows the transport of glycol-CuInS_2_ QDs inside tumor spheroids at different time points, and a time-dependent penetration of glycol-CuInS_2_ QDs in multicellular tumor spheroids can been observed from 20 to 180 min. At 20 min post-treatment, red fluorescent spots in the confocal images indicate a distribution of glycol-CuInS_2_ QDs in the outer region of the 3D model. As the time increases, enhanced fluorescence signal in the inner region can be observed, suggesting more homogeneous uptake. Finally, there glycol-CuInS_2_ QDs penetrated almost to the center of the 3D spheroids after 180 min of incubation. Besides, as shown in Fig. 9b, the fluorescent signals of Gal-CuInS_2_ and Man-CuInS_2_ QDs are observed dispersed evenly throughout tumor cells, indicating the penetration capacities are higher than those of Fru-CuInS_2_ and Glu-CuInS_2_ QDs. Previous studies have shown that the internalization ability of QDs to HeLa cellular spheroids is affected by the QDs surface charges. In general, positively-charged QDs cannot reach the necrotic zone since they can be absorbed by the proliferating cells of the spheroid. While the negatively-charged QDs can diffuse more quickly, allowing them to penetrate deeply into the tissues [56, 60]. Thus, our results are in excellent agreement with the experimental results reported in the previous study. Figure 9c shows the schematic representation of glyco-CuInS_2_ QDs penetration in 3D MKN-45 spheroid. Glyco-CuInS_2_ QDs can succeed in penetrating uniformly into the interior of 3D MCTS and reach the necrotic zone due to their high negative charge (zeta potential values ranging from − 23.9 to − 30.1 mV). which overcame the problem of poor penetration depth of existing QDs in in vitro spheroid models. These results indicate that glycol-CuInS_2_ QDs have excellent tumor penetration capacity, which is of particular importance since the fluorescence probes used for cancer imaging must efficiently penetrate tumor tissues to reach all of the viable cells [61, 62]. Therefore, these glycol-CuInS_2_ QDs have great potential to be used as a fluorescent probe for intratumoral analysis.

### The dual-color bioimaging of glycol-CuInS_2_ QDs in zebrafish

**Fig. 10:**
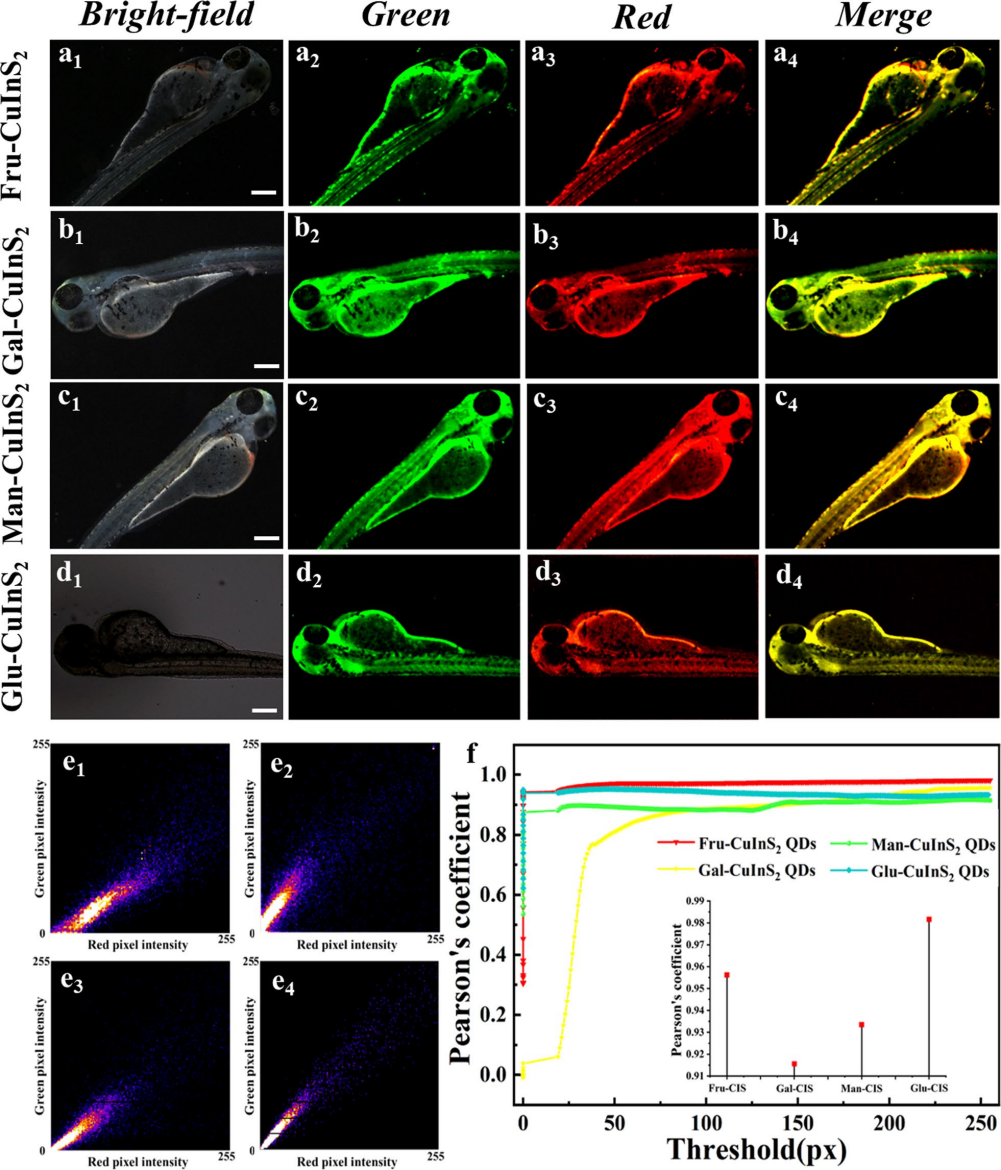
Microscopic images of two channels of zebrafish after 1 h incubation with glyco-CuInS_2_ QDs. **a**_**1**_–**d**_**1**_ bright field; **a**_**2**_–**d**_**2**_ green channel; **a**_**3**_–**d**_**3**_ red channel; **a**_**4**_–**d**_**4**_ merged. Scale bars are 600 μm. **e**_**1**_–**e**_**4**_ Pixel plots of imaging confocal after treatment of zebrafish with Fru-CuInS_2_, Gal-CuInS_2_, Man-CuInS_2_ and Glu-CuInS_2_ QDs samples; (**f**) Plot of threshold versus Pearson's correlation coefficient in fluorescence confocal detection (inset: four samples treated with zebrafish overlap of the red and green channel imaging positions)

Finally, we investigated the imaging abilities of glycol-CuInS_2_ QDs in living organisms. Based on the genetic similarity of 87% to humans, the zebrafish has outstanding advantages as a model organism because its experimental.results are applicable to humans in most cases. Nowadays, zebrafish has become one of the most valuable models in vertebrate developmental biology and shows great potential for use in other disciplines [63]. Thus, using zebrafish, it is possible to study fundamental questions in the life sciences and to reveal the molecular mechanisms of embryonic and tissue organ development. Therefore, zebrafish is chosen as model organism in our study. Firstly, zebrafishes were incubated with glycol-CuInS_2_ QDs aqueous solution (1 μM) in a 96-well plate for 1 h. Subsequently, two-channel fluorescence signals were recorded by the fluorescence microscope. As shown in Fig. 10, all glycol-CuInS_2_ QDs in zebrafish display strong fluorescence signals in green and red channels under different excitation sources of fluorescence microscopy. The results indicate that these glycol-CuInS_2_ QDs can be absorbed through the skin of zebrafish and are low toxicity in biological systems. Moreover, we used Image J software to perform accurate calculations of the fluorescence intensity of the imaging (Additional file 1: Fig. S18) and used plug-ins to measure the fluorescence position in live imaging of different channels of zebrafish (Fig. 10e_1_-e_4_). The trends of pixel point dispersion in the QDs show the proportional overlap in the imaging of the two channels, and the overlap rate is quantified in Fig. 10f, which surprisingly remains above 91.56% for all glycol-CuInS_2_ QDs, showing a high degree of overlap. Therefore, these results clearly demonstrate that these glycol-CuInS_2_ QDs synthesized by aqueous method have great potential in in vivo bioluminescence imaging.

## Conclusions

We developed an eco-friendly green approach to the aqueous-phase synthesis of QDs-based fluorescent nanobiosensor by the “direct” reaction of thiol-ending natural monosaccharides with metal salts precursors. Compared with existing synthesis methods through surface modification of QDs by carbohydrate, the “direct” strategy is simpler and more low-cost. The formation of glyco-CuInS_2_ QDs could be explained by a nucleation-growth mechanism following the LaMer model. As-prepared glyco-CuInS_2_ QDs (Fru-CuInS_2_, Gal-CuInS_2_, Man-CuInS_2_ and Glu-CuInS_2_ QDs) showed monodisperse spherical morphology (< 5 nm), good water solubility and non-cytotoxicity. These glyco-QDs exhibited excitation-wavelength-dependent visible/NIR dual emission with good photostablity and favorable photoreversibilit, which may be attributable to visible excitonic emission and near-infrared surface defect emission, respectively. Confocal microscopy experiments showed that glyco-QDs had highly specific targeting ability for cancer cell membranes (HeLa, A549, MKN-45) due to their good biorecognition ability originating from glycocluster on the surface of the CuInS_2_ QDs. Therefore, these glyco-QDs can be used as a potential plasma membrane imaging reagent for dual-color (green and red) imaging of cell membrane. More importantly, these glyco-QDs could succeed in penetrating uniformly into the interior of 3D multicellular tumor spheroids (MCTS) because of their high negative charge, thus suggesting the possibility to exploit such nanoprobes for the tumor bioimaging with excellent tumor penetration capacity. Therefore, our prepared glyco-QDs shows great commercial prospects in the field of biomedicine due to their low cost, simple manufacturing process and deep tumor penetration capacity. Besides, these glyco-CuInS_2_ QDs can exhibit more efficient recognition of tumor cells by coating with tumor-targeting agents such as cRGD.

## Supplementary Information

**Additional file 1: Figure S1** 1H NMR hydrogen spectra of (a) Fru-CuInS_2_ QDs, (b) Gal-CuInS_2_ QDs, (c) Man-CuInS_2_ QDs and (d) Glu-CuInS_2_ QDs (room temperature, D_2_O). **Figure S2.** MS spectra of (a) Fru-CuInS_2_ QDs, (b) Gal-CuInS_2_ QDs, (c) Man-CuInS_2_ QDs and (d) Glu-CuInS_2_ QDs. **Figure S3.** Fluorescence emission spectra of (a) Fru-CuInS_2_ QDs, (b) Gal-CuInS_2_ QDs, (c) Man-CuInS_2_ QDs, and (d) Glu-CuInS_2_ QDs synthesized at different dose ratios. (Inset: relationship between dose ratio and luminescence intensity of CuInS_2_ QDs). **Figure S4.** Fluorescence emission spectra of (a) Fru-CuInS_2_ QDs, (b) Gal-CuInS_2_ QDs, (c) Man-CuInS_2_ QDs, and (d) Glu-CuInS_2_ QDs synthesized under different pH conditions. (Inset: relationship between pH and luminescence intensity of CuInS_2_ QDs). **Figure S5.** Fluorescence emission spectra of (a) Fru-CuInS_2_ QDs, (b) Gal-CuInS_2_ QDs, (c) Man-CuInS_2_ QDs, and (d) Glu-CuInS_2_ QDs synthesized under different temperature conditions. (Inset: Relationship between temperature and luminescence intensity of CuInS_2_ QDs). **Figure S6.** The SEM–EDS analysis of Fru-CuInS_2_, Gal-CuInS_2_, Man-CuInS_2_ and Glu-CuInS_2_ QDs. **Figure S7.** The EDX elemental mapping of Fru-CuInS_2_, Gal-CuInS_2_, Man-CuInS_2_ and Glu-CuInS_2_ QDs. **Figure S8.** The XPS survey spectra of (a)Fru-CuInS_2_ QDs, (b)Gal-CuInS_2_ QDs, (c)Man-CuInS_2_ QDs, and (d)Glu-CuInS_2_ QDs. **Figure S9.** Zeta potential values of glyco-CuInS_2_ QDs. **Figure S10.** TEM images of Fru-CuInS_2_, Gal-CuInS_2_, Man-CuInS_2_ and Glu-CuInS_2_ QDs at a scale of 5 nm after fourteen days. **Figure S11.** DOS and PDOS of Fructose molecules adsorbed on CuInS_2_(001) before and after (a) DOS of CuInS_2_(001) system, (b) Fructose molecules adsorbed on DOS of CuInS_2_(001) system, (c) PDOS of CuInS_2_(001) system, (d) Fructose molecules adsorbed on DOS of CuInS_2_(001) system. **Figure S12.** The UV–Vis absorption spectra of Fru-CuInS_2_, Gal-CuInS_2_, Man-CuInS_2_ and Glu-CuInS_2_ QDs, respectively. **Figure S13.** Fluorescence lifetimes of glyco-CuInS_2_ QDs. **Figure S14.** Excitation and emission fluorescence spectra of (a) Fru-CuInS_2_ QDs, (b) Gal-CuInS_2_ QDs, (c) Man-CuInS_2_ QDs, and (d) Glu-CuInS_2_ QDs. **Figure S15.** Fluorescence stabilities of (a) Fru-CuInS_2_ QDs, (b) Gal-CuInS_2_ QDs, (c) Man-CuInS_2_ QDs and (d) Glu-CuInS_2_ QDs in solution. The black, red and blue lines refer to the fluorescence spectra of glyco-CuInS_2_ QDs after 0, 7, and 14 days, respectively. **Figure S16.** Fluorescence spectra of the pH response of (a) Fru-CuInS_2_ QDs, (b) Gal-CuInS_2_ QDs, (c) Man-CuInS_2_ QDs, and (d) Glu-CuInS_2_ QDs. (Inset: relationship between pH and luminescence intensity of CuInS_2_ QDs). **Figure S17.** MTT assay to detect changes in cellular activity of cancer cells after the addition of 20,40,60,80,100 μg/mL of (a) Fru-CuInS_2_ QDs, (b) Gal-CuInS_2_ QDs, (c) Man-CuInS_2_ QDs, and (d) Glu-CuInS_2_ QDs, respectively. **Figure S18.** Fluorescence intensities of (a)Fru-CuInS_2_ QDs, (b) Gal-CuInS_2_ QDs, (c) Man-CuInS_2_ QDs, and(d) Glu-CuInS_2_ QDs imaged under fluorescence microscopy after incubation of zebrafish for 1 h, respectively. **Table S1.** Exploration of optimal synthesis conditions for glyco-CuInS_2_ QDs. **Table S2.** The photophysical properties of glyco-CuInS_2_ QDs.

## References

[1] Nasrollahpour H, Khalilzadeh B, Hasanzadeh M, Rahbarghazi R, Estrela P, Naseri A, Tasoglu S, Sillanpää M (2022). Nanotechnology-based electrochemical biosensors for monitoring breast cancer biomarkers. Med Res Rev.

[2] Pourmadadi M, Rahmani E, Rajabzadeh-Khosroshahi M, Samadi A, Behzadmehr R, Rahdar A, Ferreira LFR (2023). Properties and application of carbon quantum dots (CQDs) in biosensors for disease detection: a comprehensive review. J Drug Deliv Sci Tec.

[3] Sha Q, Fei J, Tu C, Liu B, Hu Z, Liu X (2022). A universal strategy of glyconanoparticle preparation using a bifunctional linker for lectin sensing and cell imaging. Microchim Acta.

[4] Hao N, Neranon K, Ramström O, Yan M (2016). Glyconanomaterials for biosensing applications. Biosens Bioelectron.

[5] Norouzi F, Pourmadadi M, Yazdian F, Khoshmaram K, Mohammadnejad J, Sanati MH, Chogan F, Rahdar A, Baino F (2022). PVA-based nanofibers containing chitosan modified with graphene oxide and carbon quantum dot-doped TiO_2_ enhance wound healing in a rat model. J Funct.

[6] Tang Q, Huang G (2018). Preparation and applications of glyconanoparticles. Int J Biol Macromol.

[7] Zhang X, Huang G, Huang H (2019). The glyconanoparticle as carrier for drug delivery. Drug Deliv.

[8] Fuente JMDL, Barrientos AG, Rojas TC, Rojo J, Canada J, Frenandez A, Penades S (2001). Gold glyconanoparticles as water-soluble polyvalent models to study carbohydrate interactions. Angew Chem Int Edit.

[9] Kim J, Hwang DW, Jung HS, Kim KW, Pham XH, Lee SH, Byun JW, Kim W, Kim HM, Hahm E (2022). High-quantum yield alloy-typed core/shell CdSeZnS/ZnS quantum dots for bio-applications. J Nanobiotechnol.

[10] Aires A, Möller M, Cortajarena AL (2020). Protein design for the synthesis and stabilization of highly fluorescent quantum dots. Chem Mater.

[11] Pourmadadi M, Moammeri A, Shamsabadipour A, Moghaddam YF, Rahdar A, Pandey S (2023). Application of various optical and electrochemical nanobiosensors for detecting cancer antigen 125 (CA-125): a review. Biosensors.

[12] Ashree J, Wang Q, Chao Y (2020). Glyco-functionalised quantum dots and their progress in cancer diagnosis and treatment. Front Chem Sci Eng.

[13] Pourmadadi M, Rajabzadeh-Khosroshahi M, Saeidi Tabar F, Ajalli N, Samadi A, Yazdani M, Yazdian F, Rahdar A, Díez-Pascual AM (2022). Two-dimensional grap hitic carbon nitride (g-C_3_N_4_) nanosheets and their derivatives for diagnosis and detection applications. J Funct.

[14] Ohyanagi T, Nagahori N, Shimawaki K, Hinou H, Yamashita T, Sasaki A, Jin T, Iwanaga T, Kinjo M, Nishimura SI (2011). Importance of sialic acid residues illuminated by live animal imaging using phosphorylcholine self-assembled monolayer-coated quantum dots. J Am Chem Soc.

[15] Biagiotti G, Angeli A, Giacomini A, Toniolo G, Landini L, Salerno G, Di Cesare ML, Ghelardini C, Mello T, Mussi S, Ravelli C, Marelli M, Cicchi S, Menna E, Ronca R, Supuran CT, Richichi B (2021). Glyco-coated CdSe/ZnS quantum dots as nanoprobes for carbonic anhydrase IX imaging in cancer cells. ACS Appl Nano Mater.

[16] Allocca M, Mattera L, Bauduin A, Miedziak B, Moros M, De Trizio L, Tino A, Reiss P, Ambrosone A, Tortiglione C (2019). An integrated multilevel analysis profiling biosafety and toxicity induced by indium- and cadmium-based quantum dots in vivo. Environ Sci Technol.

[17] Nikazar S, Sivasankarapillai VS, Rahdar A, Gasmi S, Anumol PS, Shanavas MS (2020). Revisiting the cytotoxicity of quantum dots: an in-depth overview. Biophys Rev.

[18] Zikalala N, Parani S, Tsolekile N, Oluwafemi OS (2020). Facile green synthesis of ZnInS quantum dots: temporal evolution of their optical properties and cell viability against normal and cancerous cells. J Mater Chem C.

[19] Wang X, Tian J, Yong KT, Zhu X, Lin MCM, Jiang W, Li J, Huang Q, Lin G (2016). Immunotoxicity assessment of CdSe/ZnS quantum dots in macrophages, lymphocytes and BALB/c mice. J Nanobiotechnol.

[20] Zhang Y, Qiao L, Zhang Z, Liu Y, Li L, Shen H, Zhao M (2022). A mitochondrial-targetable fluorescent probe based on high-quality InP quantum dots for the imaging of living cells. Mater Des.

[21] Ding K, Jing L, Liu C, Hou Y, Gao M (2014). Magnetically engineered Cd-Free quantum dots as dual-modality probes for fluorescence/magnetic resonance imaging of tumors. Biomaterials.

[22] Liu Z, Liu H, Wang L, Su X (2016). A label-free fluorescence biosensor for highly sensitive detection of lectin based on carboxymethyl chitosan-quantum dots and gold nanoparticles. Anal Chim Acta.

[23] Marradi M, Tricomi J, Matassini C, Richichi B, Marco M (2021). Carbohydrate functionalized quantum dots in sensing, imaging and therapy applications. Comprehensive Glycoscience.

[24] Jesús M, Penadés S (2005). Glyco-quantum dots: a new luminescent system with multivalent carbohydrate display. Tetrah Asym.

[25] Krejcova L, Nejdl L, Rodrigo MM, Zurek M, Matousek M, Hynek D, Zitka O, Kopel P, Adam V, Kizek R (2013). 3D printed chip for electrochemical detection of influenza virus labeled with CdS quantum dots. Biosens.

[26] Liu W, Greytak AB, Lee J, Wong CR, Park J, Marshall LF, Jiang W, Curtin PN, Ting AY, Nocera DG (2010). Compact biocompatible quantum dots via RAFT-mediated synthesis of imidazole-based random copolymer ligand. J Am Chem Soc.

[27] Lima CN, Oliveira WF, Silva PMM, Filho PEC, Juul-Madsen K, Moura P, Vorup-Jensen T, Fontes A (2022). Mannose-binding lectin conjugated to quantum dots as fluorescent nanotools for carbohydrate tracing. Methods Appl Fluoresc.

[28] Branzi L, Purcell-Milton F, Cressoni C, Back M, Cattaruzza E, Speghini A, Gun'ko YK, Benedetti A (2022). Chiral non-stoichiometric ternary silver indium sulfide quantum dots: investigation on the chirality transfer by cysteine. Nanoscale.

[29] Arshad A, Chen H, Bai X, Xu S, Wang L (2016). One-pot aqueous synthesis of highly biocompatible near infrared CuInS_2_ quantum dots for target cell imaging. Chinese J Chem.

[30] Xiong W, Yang G, Wu X, Zhu J (2013). Aqueous synthesis of color-tunable CuInS_2_/ZnS nanocrystals for the detection of human interleukin 6. ACS Appl Mater.

[31] Guan X, Yang X, Lai S, Ding Y, Wei J, Zhang J, Zhang L, Li C, Tong J, Lei Z (2022). Design and synthesis of biodegradable nonconjugated SSPAMAM dendrimers with unexpected deep-red/NIR emission and cell membrane targeting ability for biological imaging. Mater Des.

[32] Ding H, Zhang P, Wang T, Kong J, Xiong H (2014). Nitrogen-doped carbon dots derived from polyvinyl pyrrolidone and their multicolor cell imaging. Nanotechnology.

[33] Viltres H, Odio OF, Biesinger MC, Montiel G, Borja R, Reguera E (2020). Preparation of amine- and disulfide-containing PAMAM-based dendrons for the functionalization of hydroxylated surfaces: XPS as structural sensor. ChemistrySelect.

[34] Zou W, Ma X, Zheng P (2019). Preparation and functional study of cellulose/carbon quantum dot composites. Cellulose.

[35] Liu J, Zhao X, Xu H, Wang Z, Dai Z (2019). Amino acid-capped water-soluble near-infrared region CuInS_2_/ZnS quantum dots for selective cadmium ion determination and multicolor cell imaging. Anal Chem.

[36] Liu Z, Liu H, Liu L, Su X (2016). Aptamer based lysozyme assay using fluorescent CuInS_2_ quantum dots and graphene oxide, and its application to inhibitor screening. Microchim Acta.

[37] Prathna TC, Chandrasekaran N, Mukherjee A (2011). Studies on aggregation behaviour of silver nanoparticles in aqueous matrices: effect of surface functionalization and matrix composition. Colloids Surf.

[38] Delley B (1990). An all-electron numerical method for solving the local density functional for polyatomic molecules. J Chem Phys.

[39] Hammer B, Hansen LB, Nørskov JK (1999). Improved adsorption energetics within density-functional theory using revised perdew-burke-ernzerhof functionals. Phys Rev B.

[40] Perdew JP, Burke K, Ernzerhof M (1996). Generalized gradient approximation made simple. Phys Rev Lett.

[41] Sun M, Peng Y (2014). Study on structural, electronic and magnetic properties of Sn atom adsorbed on defective graphene by first-principle calculations. Appl Surf Sci.

[42] Yao J, Li P, Li L, Yang M (2018). Biochemistry and biomedicine of quantum dots: from biodetection to bioimaging, drug discovery, diagnostics, and therapy. Acta Biomater.

[43] Jiang T, Song J, Wang H, Ye X, Wang H, Zhang W, Yang M, Xia R, Zhu L, Xu X (2015). Aqueous synthesis of color tunable Cu doped Zn-In-S/ZnS nanoparticles in the whole visible region for cellular imaging. J Mater Chem B.

[44] Arshad A, Akram R, Iqbal S, Batool F, Iqbal B, Khalid B, Khan AU (2019). Aqueous synthesis of tunable fluorescent, semiconductor CuInS_2_ quantum dots for bioimaging. Arab J Chem.

[45] Guan X, Lu B, Jin Q, Li Z, Wang L, Wang K, Lai S, Lei Z (2018). AIE-active fluorescent nonconjugated polymer dots for dual-alternating-color live cell imaging. Ind Eng Chem Res.

[46] Guan X, Li Z, Wang L, Liu M, Lei Z (2019). Preparation of AIE polymer dots (Pdots) based on poly (N-vinyl-2-pyrrolidone)-Eu (III) complex and dual-color live cell imaging. Acta Chim Sinica.

[47] Sowers KL, Swartz B, Krauss TD (2013). Chemical mechanisms of semiconductor nanocrystal synthesis. Chem Mater.

[48] Wang X, Liang Z, Xu X, Wang N, Fang J, Wang J, Xu G (2015). A high efficient photoluminescence Zn–Cu–In–S/ZnS quantum dots with long lifetime. J Alloys Compd.

[49] Zaiats G, Kinge S, Kamat PV (2016). Origin of dual photoluminescence states in ZnS–CuInS_2_ alloy nanostructures. J Phys Chem C.

[50] Jara DH, Yoon SJ, Stamplecoskie KG, Kamat PV (2014). Size-dependent photovoltaic performance of CuInS_2_ quantum dot-sensitized solar cells. Chem Mater.

[51] Jara DH, Stamplecoskie KG, Kamat PV (2016). Two distinct transitions in Cu_x_InS_2_ quantum dots. bandgap versus sub-bandgap excitations in copper-deficient structures. J Phys Chem Lett.

[52] Jing X, Hu H, Sun Y, Yu B, Cong H, Shen Y (2022). The intracellular and extracellular microenvironment of tumor site: the trigger of stimuli-responsive drug delivery systems. Small Methods.

[53] Zhang S, Zhang D, Ding Y, Hua J, Tang B, Ji X, Zhang Q, Wei Y, Qin K, Li B (2019). Bacteria-derived fluorescent carbon dots for highly selective detection of p-Nitrophenol and bioimaging. Analyst.

[54] Maddalena S, Walter P, Anna B, Antonella P, Markus D, Lorenza M, Rita C (2022). Manganese in diagnostics: a preformulatory study. Pharmaceutics.

[55] Arena ET, Rueden CT, Hiner MC, Wang S, Yuan M, Eliceiri KW (2017). Quantitating the cell: turning images into numbers with ImageJ. Wires Dev Biol.

[56] Liu Y, Ji D, Dong L, Galanos N, Zang Y, Li J, Vidal S, He X (2017). Supramolecular assembly of fluorogenic glyco-dots from perylenediimide-based glycoclusters for targeted imaging of cancer cells. ChemComm.

[57] Wang D, Su H, Kwok RT, Hu X, Zou H, Luo Q, Lee MMS, Xu W, Lam JWY, Tang BZ (2018). Rational design of a water-soluble NIR AIEgen, and its application in ultrafast wash-free cellular imaging and photodynamic cancer cell ablation. Chem Sci.

[58] Naya G, Joseph BW, Paul RM, Edward JS, Johannes PM, Iestyn P, Lukas P, Cameron A, Arwyn TJ, Wolfgang L, Peter W, Paola B (2020). Four-wave-mixing microscopy reveals non-colocalisation between gold nanoparticles and fluorophore conjugates inside cells. Nanoscale.

[59] Xu W, Lee M, Nie J, Zhang Z, Kwok R, Lam J, Xu F, Wang D, Tang B (2020). Three-pronged attack by homologous far-red/NIR AIEgens to achieve 1+1+1>3 synergistic enhanced photodynamic therapy. Angew Chem Int Ed.

[60] Dirheimer L, Pons T, Marchal F, Bezdetnaya L (2022). Quantum dots mediated imaging and phototherapy in cancer spheroid models: state of the art and perspectives. Pharmaceutics.

[61] Przysiecka J, Michalska M, Nowaczyk G, Pepliſska B, Jesionowski T, Schneider R, Jurga S (2016). RGD peptide as effective transporter of CuInZn_x_S_2+x_ quantum dots into human cancer cells. Colloid Surface.

[62] Jiang X, Xin H, Gu J, Xu X, Xia W, Chen S, Xie Y, Chen L, Chen Y, Sha X, Fang X (2013). Solid tumor penetration by integrin-mediated pegylated poly (trimethylene carbonate) nanoparticles loaded with paclitaxel. Biomaterials.

[63] Ko SK, Chen X, Yoon J, Shin I (2011). Zebrafish A good vertebrate model for molecular imaging using fluorescent probes. Chem Soc Rev.

