# Supplementary material for: Green synthesis of glyco-CuInS2 QDs with visible/NIR dual emission for 3D multicellular tumor spheroid and in vivo imaging
Source: J Nanobiotechnology. 2023 Apr 1;21:118. doi: 10.1186/s12951-023-01859-6 (PMC10067196; doi:10.1186/s12951-023-01859-6)
Supplement: Supplementary file 1 — Additional file 1: Figure S1 1H NMR hydrogen spectra of (a) Fru-CuInS2 QDs, (b) Gal-CuInS2 QDs, (c) Man-CuInS2 QDs and (d) Glu-CuInS2 QDs (room temperature, D2O). Figure S2. MS spectra of (a) Fru-CuInS2 QDs, (b) Gal-CuInS2 QDs, (c) Man-CuInS2 QDs and (d) Glu-CuInS2 QDs. Figure S3. Fluorescence emission spectra of (a) Fru-CuInS2 QDs, (b) Gal-CuInS2 QDs, (c) Man-CuInS2 QDs, and (d) Glu-CuInS2 QDs synthesized at different dose ratios. (Inset: relationship between dose ratio and luminescence intensity of CuInS2 QDs). Figure S4. Fluorescence emission spectra of (a) Fru-CuInS2 QDs, (b) Gal-CuInS2 QDs, (c) Man-CuInS2 QDs, and (d) Glu-CuInS2 QDs synthesized under different pH conditions. (Inset: relationship between pH and luminescence intensity of CuInS2 QDs). Figure S5. Fluorescence emission spectra of (a) Fru-CuInS2 QDs, (b) Gal-CuInS2 QDs, (c) Man-CuInS2 QDs, and (d) Glu-CuInS2 QDs synthesized under different temperature conditions. (Inset: Relationship between temperature and luminescence intensity of CuInS2 QDs). Figure S6. The SEM–EDS analysis of Fru-CuInS2, Gal-CuInS2, Man-CuInS2 and Glu-CuInS2 QDs. Figure S7. The EDX elemental mapping of Fru-CuInS2, Gal-CuInS2, Man-CuInS2 and Glu-CuInS2 QDs. Figure S8. The XPS survey spectra of (a)Fru-CuInS2 QDs, (b)Gal-CuInS2 QDs, (c)Man-CuInS2 QDs, and (d)Glu-CuInS2 QDs. Figure S9. Zeta potential values of glyco-CuInS2 QDs. Figure S10. TEM images of Fru-CuInS2, Gal-CuInS2, Man-CuInS2 and Glu-CuInS2 QDs at a scale of 5 nm after fourteen days. Figure S11. DOS and PDOS of Fructose molecules adsorbed on CuInS2(001) before and after (a) DOS of CuInS2(001) system, (b) Fructose molecules adsorbed on DOS of CuInS2(001) system, (c) PDOS of CuInS2(001) system, (d) Fructose molecules adsorbed on DOS of CuInS2(001) system. Figure S12. The UV–Vis absorption spectra of Fru-CuInS2, Gal-CuInS2, Man-CuInS2 and Glu-CuInS2 QDs, respectively. Figure S13. Fluorescence lifetimes of glyco-CuInS2 QDs. Figure S14. Excitation and emission fluorescence spe [file 12951_2023_1859_MOESM1_ESM.docx]

**Supplementary**

Green Synthesis of Glyco-CuInS_2_ QDs with Visible/NIR Dual Emission for 3D Multicellular Tumor Spheroid and in Vivo Imaging

Xiaolin Guan, ^*, a, †^ Liyuan Zhang, ^a, †^ Shoujun Lai, ^*, b^ Jiaming Zhang, ^a^ Jingyu Wei, ^a^ Kang Wang, ^a^ Wentao Zhang, ^a^ Chenhao Li, ^c^ Jinhui Tong, ^a^ and Ziqiang Lei ^a^

a Key Laboratory of Eco-Environment-Related Polymer Materials Ministry of Education, Key Laboratory of Polymer Materials Ministry of Gansu Province, College of Chemistry and Chemical Engineering, Northwest Normal University, Lanzhou, Gansu 730070, P.R. China.

b College of Chemical Engineering, Lanzhou University of Arts and Science, Lanzhou, Gansu 730000, P.R. China.

c Key Laboratory of Traditional Chinese Medicine Prevention and Treatment, Gansu University of Traditional Chinese Medicine, Lanzhou 730000, China

**This PDF file includes:**

Experimental Section

Figures S1 to S18

Tables S1 to S2

Video S1 to S4

s

**Experimental**

**Materials and Methods**

D-fructose (Fru, 99%), D-galactose (Gal, 99%), D-mannose (Man, 99%), and D-glucose (Glu, 99%) were obtained from Sane Chemtech (Shanghai) Co., Ltd as a commercial product and used directly. 1-(3-dimethylaminopropyl)-3-ethylcarbodiimide hydrochloride (EDC, 99%), 3-Mercaptopropionic acid (MPA, 98%), 4-dimethylaminopyridine (DMAP, 98%) were purchased from Sane Chemtech (Shanghai) Co., Ltd and used without purification. N, N-Dimethylformamide (DMF, AR), Acetone (AR), Dimethyl Sulfoxide (DMSO, AR), and Sodium hydroxide (NaOH, 98%) were purchased from Chengdu Kolon Chemicals Co. Ltd and used without purification. Trypsin-EDTA (0.25%) was obtained from Shanghai Yuanpei Biotechnology Co. Thiazole Blue was purchased from Bio, Hyclone Bio Reagents, USA. All other chemicals not mentioned here were of analytical grade and obtained from commercial sources. Doubly distilled water was used throughout the experiments.

Fourier transform infrared (FT-IR) spectra were measured by a Nicolet AVATAR 360 spectrometer from Perkin Elmer, USA, in the range 4000-400 cm^-1^. X-ray photoelectron spectroscopy (XPS) was measured by a ThermoFisher device, model Thermo Scientific K-Alpha+. X-ray diffraction (XRD) data were obtained from a D/max-III AX-ray diffractometer from Rigaku, Japan. Transmission electron microscopy (TEM) photographs were taken by a JEM-1200EX transmission electron microscope from JEOL, Japan. The scanning electron microscopy energy dispersive spectrometry (SEM-EDS) data were obtained by a ULTRA Plus transmission electron microscope from Germany. Zeta potential was measured by a Nano-ZS90 dynamic light scattering (DLS) nanoparticle sizer from Malvern Ltd, USA. The UV-Vis and PL data were obtained by a Shimadzu UV-2550 spectrometer, Japan and a Perkin Elmer F97 Pro spectrometer, USA. Fluorescence steady-state and lifetime measured by the Edinburgh FLS1000 steady-state/transient fluorescence spectrometer. The reactor was from Lanzhou Jiatexing Co. The electrically heated constant temperature air dryer (DGH-9036A) was from Shanghai Precision Scientific Instruments Co. The cell and zebrafish imaging images were taken by a Zeiss Axio Scope.A1 ortho-fluorescence microscope from Germany. MTT method toxicity experimental data were obtained from RT-6000 enzyme labelling instrument tests. Simulation of 3D tumour models by high content analysis system (PerkinElmer, Operetta CLS).

Synthesis of thiolated monosaccharide ligands

Thiolated monosaccharide was synthesized according to the published procedure.^1-2^ Briefly, EDC (0.70 g, 3.65 mmol), 3-MPA (0.34 ml, 3.90 mmol) and DMAP (0.50 g, 4.09 mmol) were dissolved in a round bottom flask containing 100 mL of DMF at 0°C. The mixture was kept at constant temperature (0 °C) and stirred for 1 h. Afterwards, D-fructose (0.5 g, 2.78 mmol) was added to the reaction system and stirred for 24 h at room temperature. After the reaction was completed and the solution was concentrated, the precipitate was obtained by adding anhydrous acetone (1:10 by volume), centrifuged, washed and dried to obtain thiolated D-fructose (SH-Fru) in 80.1% yield. The molecular structure of SH-Fru could be confirmed by ^1^H NMR, MS and IR (FigureS1, FigureS2 and Figure1). ^1^H NMR (600 MHz, D_2_O) δ: 5.04 (d, *J* = 7.8 Hz, 1H), 4.59 (t, *J* = 6.7 Hz, 3H), 4.38 (t, *J* = 10.4 Hz, 2H), 4.32-4.29 (m, 1H), 4.20 (t, *J* = 7.2 Hz, 1H), 3.71-3.60 (m, 3H), 2.88-2.84 (m, 2H), 2.71-2.65 (m, 2H),1.11 (s, 1H) ppm. HR-MS: m/z calcd for C_9_H_16_O_7_S [M+Cl]^-^, 303.03, found:303.0272.

The preparation procedure of SH-Gal was similar to that of SH-Fru. It should be noted that D-galactose was used for the synthesis of SH-Gal, which was obtained as a white solid with a yield of 78.56%. The molecular structure of SH-Gal could be confirmed by ^1^H NMR, MS and IR (FigureS1, FigureS2 and Figure1). ^1^H NMR (600 MHz, D_2_O) δ: 5.14 (d, *J* = 7.3 Hz, 1H), 4.51 (d, *J* = 8.7 Hz, 2H), 4.49 (s, 1H), 4.17 (t, *J* = 7.7 Hz, 2H), 4.08 (t, *J* = 7.2 Hz, 1H), 3.97 (d, *J* = 10.6 Hz, 1H), 3.86-3.76 (m, 3H), 2.84 (t, *J* = 7.0 Hz, 2H), 2.69 (t, *J* = 7.3 Hz, 2H),1.14 (s, 1H) ppm. HR-MS: m/z calcd for C_9_H_16_O_7_S [M+Cl]^-^, 303.03, found: 303.0272.

The preparation procedure of SH-Man was similar to that of SH-Fru. It should be noted that D-mannose was used for the synthesis of SH-Man, which was obtained as a white solid with a yield of 77.2%. The molecular structure of SH- Man could be confirmed by^1^H NMR, MS and IR (FigureS1, FigureS2 and Figure1). ^1^H NMR (600 MHz, D_2_O) δ: 5.71 (d, *J* = 5.1 Hz, 2H), 4.68 (t, *J* = 7.0 Hz, 2H), 4.59 (s, 1H), 4.52 (d, *J* = 7.2 Hz, 1H), 3.81-3.69 (m, 2H), 3.59 (t, *J* = 10.1 Hz, 2H), 3.48 (d, *J* = 10.5 Hz, 1H), 2.80 (t, *J* = 8.7 Hz, 2H), 2.72-2.63 (m, 2H), 1.12 (s, 1H) ppm. HR-MS: m/z calcd for C_9_H_16_O_7_S [M+Cl]^-^, 303.03, found: 303.0271.

The preparation procedure of SH-Glu was similar to that of SH-Fru. It should be noted that D-glucose was used for the synthesis of SH-Glu, which was obtained as a white solid with a yield of 69.25%. The molecular structure of SH- Glu could be confirmed by ^1^H NMR, MS and IR (FigureS1, FigureS2 and Figure1). ^1^H NMR (600 MHz, D_2_O) δ: 5.64 (d, *J* = 8.5 Hz, 1H), 4.70 (s, 1H), 4.77 (s, 1H), 4.61 (d, *J* = 6.2 Hz, 1H), 4.29 (d, *J* = 10.4 Hz, 2H), 4.20 (s, 1H), 4.15 (d, *J* = 10.1 Hz, 1H), 3.78 (t, *J* = 8.9 Hz, 1H), 3.78-3.70 (m, 1H), 3.62 (d, *J* = 5.4 Hz, 1H), 2.66 (t, *J* = 7.4 Hz, 2H), 2.48 (t, *J* = 6.4 Hz, 2H), 1.05 (s, 1H) ppm. HR-MS: m/z calcd for C_9_H_16_O_7_S [M+Cl]^-^, 303.03, found: 303.0272.

**Synthesis of glyco-CuInS_2_ QDs**

First, SH-Fru (1.448 g, 5.40 mmol) prepared above was dissolved in 10.5 mL of distilled water and stirred for 20 min to been dissolved. CuCl_2_·2H_2_O (0.026 g, 0.15 mmol) and InCl_3_·4H_2_O (0.044 g, 0.15 mmol) were added to the above solution and charged with N_2_ for 30 min at room temperature to change the solution from light yellow to light green. The pH was then adjusted to 11 with NaOH solution (2.50 mol/L), during which the solution changed from light green to brownish yellow. After stirring for 10 min, CS(NH_2_)_2_ (0.0228 g, 0.30 mmol) was added to the mixed solution and was stirred at room temperature for 40 min (the precursors molar ratio of Cu:In:CS(NH_2_)_2_:SH-Fru was 1:1:2:36). Then, the reaction solution was finally transferred into a PTFE-lined stainless-steel reactor (15 mL) and treated at 150 ℃ for 18 h. The reactor was then removed, allowed to cool naturally to room temperature, and filtered to obtain brown Fru-CuInS_2_ QDs liquid. The product was collected by means of centrifugation at 20000 r/min for 10 min. The final product was dispersed in deionized water (1.0 mL) by ultrasonication and stored for later use.

The preparation procedures of Gal-CuInS_2,_ Man-CuInS_2_ and Glu-CuInS_2_ QDs were similar to that of Fru-CuInS_2_ QDs. It should be noted that SH-Gal, SH-Man and SH-Glu were used for the synthesis of the above three QDs, respectively. The optimum molar ratio of CuCl_2_·2H_2_O, InCl_3_·4H_2_O, CS(NH_2_)_2_, and SH-Gal were 1:1:2:12, 1:1:2:36 and 1:1:2:24. The molecular structures and micromorphologies of the four glyco-CuInS_2_ QDs could be confirmed by IR, XRD, XPS and TEM (Figure1and Figure2).

**Cell cytotoxicity assay and cell imaging**

**Cell culture.** The cell lines used: cervical cancer cells (HeLa), lung cancer human alveolar basal epithelial cells (A549), human gastric cancer cells (MKN-45) were provided by the School of Basic Medicine, Gansu University of Traditional Chinese Medicine.

The desired cell lines were resuscitated and cultured in a 37°C, 5% CO_2_ incubator. Cell growth was regularly observed under a microscope and cell passages were used according to cell morphology and density. PBS was added for 2 to 3 washes, followed by digestion with 0.25% trypsin. After termination of digestion, culture medium containing 12% FBS was added to disperse the cells in it. The final uniformly dispersed cytosol was sealed and continued in the incubator.

**Cell viability studies using MTT assays.** Cells in passages were tested for toxicity using the traditional MTT assay.^3,4^ Cervical cancer cells (HeLa), lung cancer human alveolar basal epithelial cells (A549), human gastric cancer cells (MKN-45), and human embryonic lung cells (MRC-5) were first passaged in cell culture medium containing 12% FBS and set aside, and when the cells grew well, the cells were transferred into 96-well plates at 2×10^4^ cells/mL, respectively, and PBS was added to the cell edge wells as a control. After 24 hours of growth in the appropriate culture medium, each cell type was treated with Fru-CuInS_2_, Gal-CuInS_2_, Man-CuInS_2_ and Glu-CuInS_2_ QDs at concentrations of 20, 40, 60, 80, and 100 μg/mL, respectively. After spiking at 37°C with 5% CO_2_ for 12-24 hours, 20 μL of MTT stock solution (5 mg/mL) was added to each well to continue incubation for 4 hours and 150 μL of DMSO was added to dissolve the precipitate. Finally, absorbance was obtained using a Spectra MAX 340PC plate reader and used to calculate relative cell viability.

Cell viability (%) = (ODs-OD_b_)/(OD_c_-OD_b_) ×100%

with s-sample, b-blank and c-control. All samples were repeated three times in parallel and the mean was taken (n = 3).^5^

**Cellular imaging.** For cell imaging experiments, HeLa, A549, MKN-45 and MRC-5 were firstly incubated in a culture medium containing 12% fetal bovine serum at 37°C and 5% CO_2_ respectively until they were in good morphology and at moderate density, then plated in 12-well plates and continued to grow for 24 hours. A 100 μL sample of glyco-CuInS_2_ QDs at a concentration of 100 μg/mL was incubated again with the cytosol. Cells were incubated in the same manner at different incubation times of 2 h, 6 h and 12 h, respectively, and observed and photographed under a Zeiss Axio Scope. A1 fluorescence microscope.

**3D tumor model construction and monitoring.** U-bottom 3D cell culture plates (PerkinElmer, spheroid ULA/CS, CellCarrier-96) were used to establish the microsphere model. MKN-45 cells were seeded in a 96-well plate with a U bottom (1×10^3^ cells/well). After the microspheres became visible to the naked eye, cells were treated with glyco-CuInS_2_ QDs and analyzed with a highcontent analysis system (PerkinElmer, Operetta CLS).

Real-time, glyco-CuInS_2_ QDs were monitored with a high content analysis system. Under bright field and 647nm wavelength conditions, three-Dimensional (3D) Microspheres were dynamically monitored by a content analyzer for 3 h.

**Zebrafish imaging.** First, the incubated zebrafish were kept at 28°C for 2-3 days, washed with distilled water and then glyco-CuInS_2_ QDs were added separately at a test concentration of 1 μM. After incubation in the samples for 2 h, the samples were rinsed 2-3 times with PBS buffer, placed on slides and images of zebrafish were obtained using a Zeiss Axio Scope.A1 fluorescence microscope.


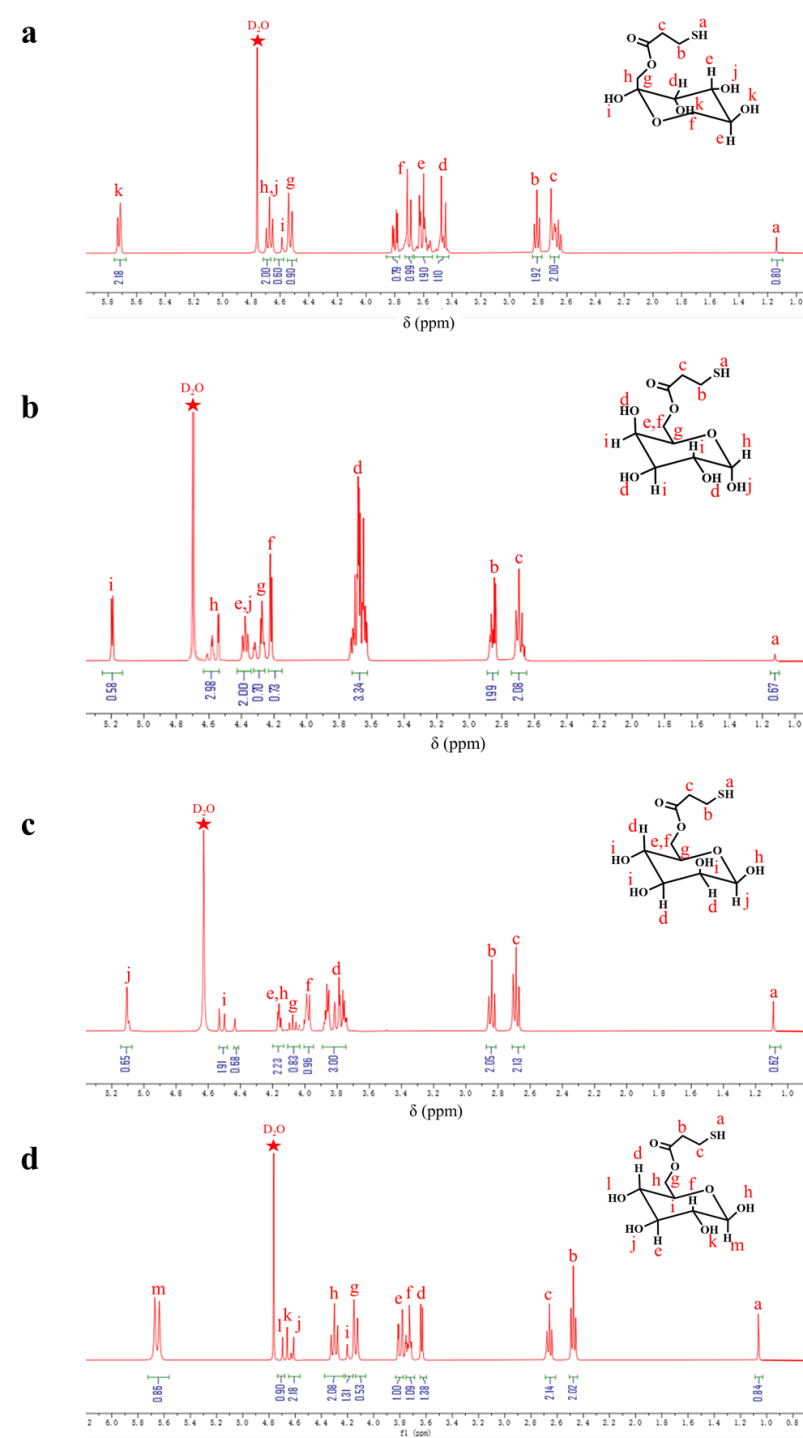


**Figure S1** 1H NMR hydrogen spectra of (a) Fru-CuInS_2_ QDs, (b) Gal-CuInS_2_ QDs, (c) Man-CuInS_2_ QDs and (d) Glu-CuInS_2_ QDs (room temperature, D_2_O).


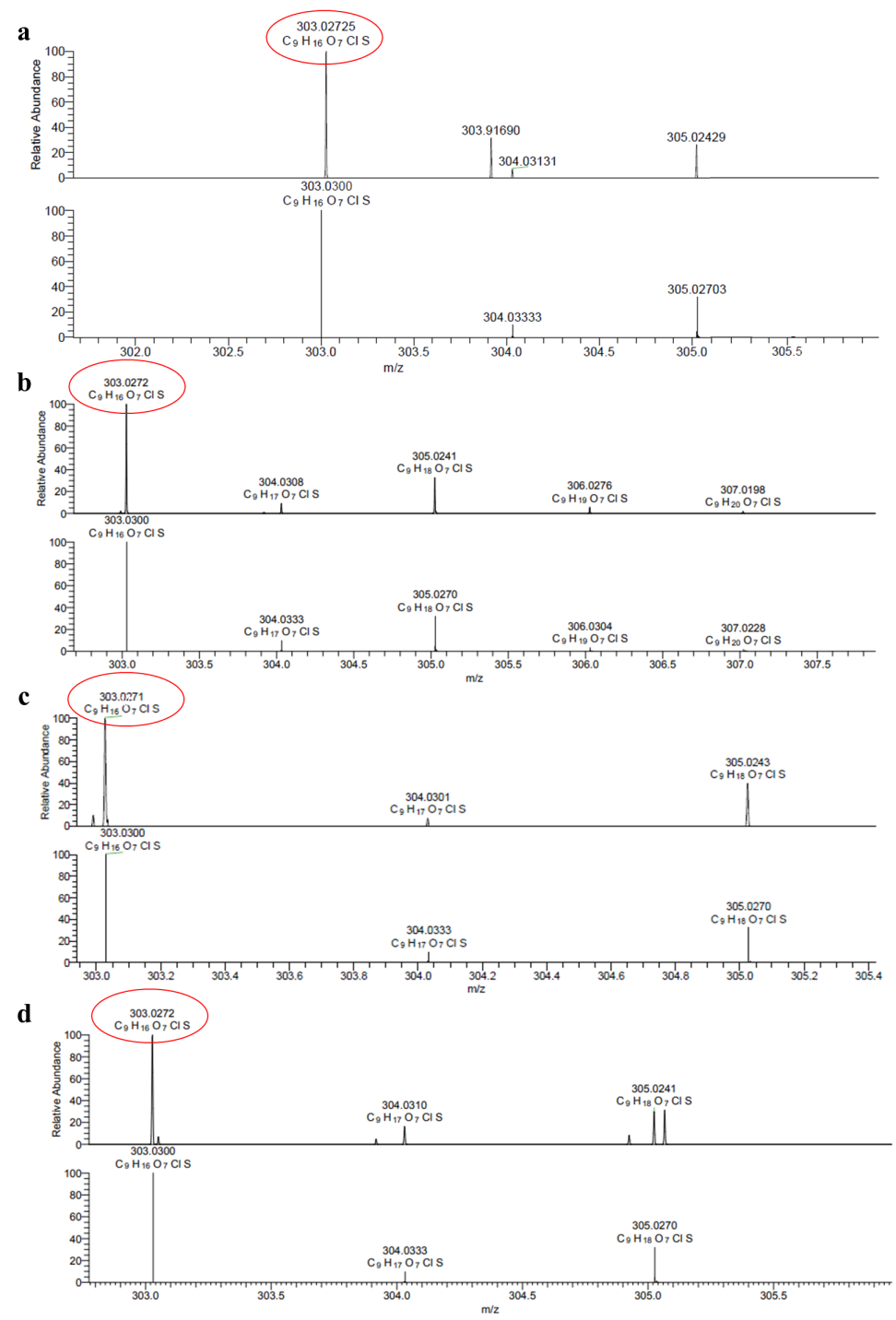


**Figure S2** MS spectra of (a) Fru-CuInS_2_ QDs, (b) Gal-CuInS_2_ QDs, (c) Man-CuInS_2_ QDs and (d) Glu-CuInS_2_ QDs.


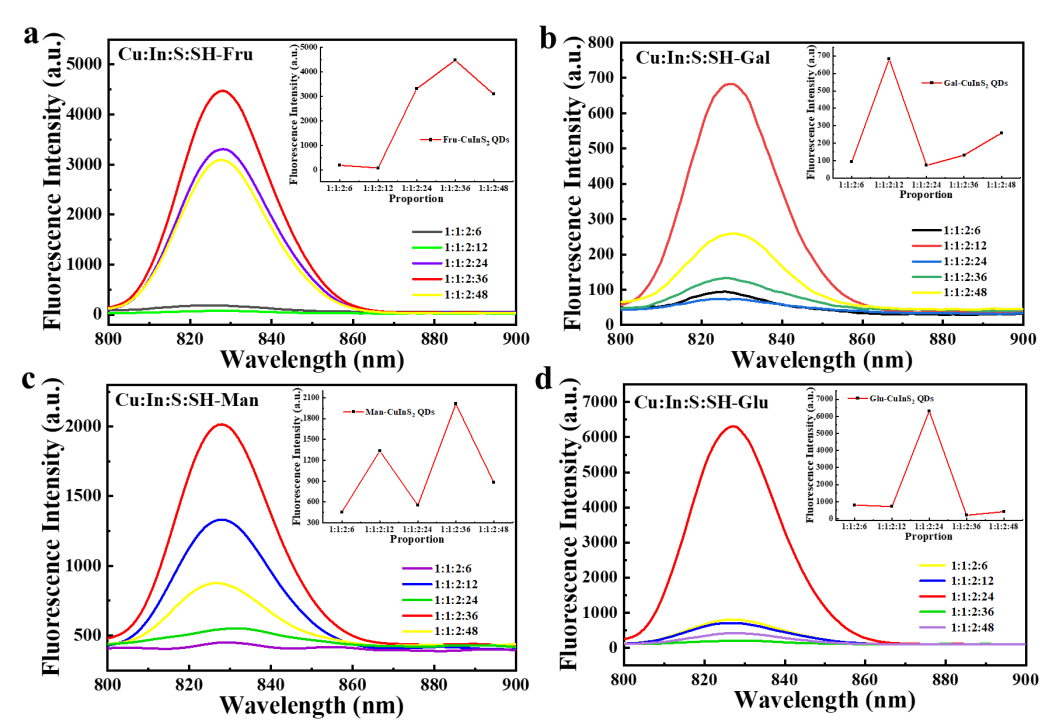


**Figure S3** Fluorescence emission spectra of (a) Fru-CuInS_2_ QDs, (b) Gal-CuInS_2_ QDs, (c) Man-CuInS_2_ QDs, and (d) Glu-CuInS_2_ QDs synthesized at different dose ratios. (Inset: relationship between dose ratio and luminescence intensity of CuInS_2_ QDs).


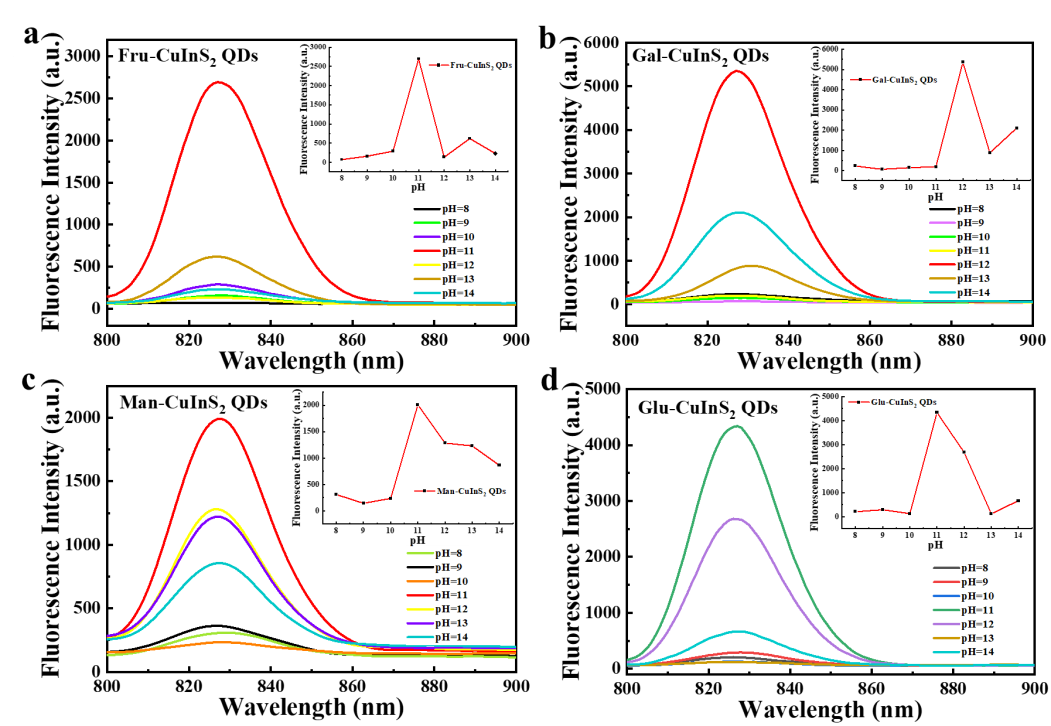


**Figure S4** Fluorescence emission spectra of (a) Fru-CuInS_2_ QDs, (b) Gal-CuInS_2_ QDs, (c) Man-CuInS_2_ QDs, and (d) Glu-CuInS_2_ QDs synthesized under different pH conditions. (Inset: relationship between pH and luminescence intensity of CuInS_2_ QDs).


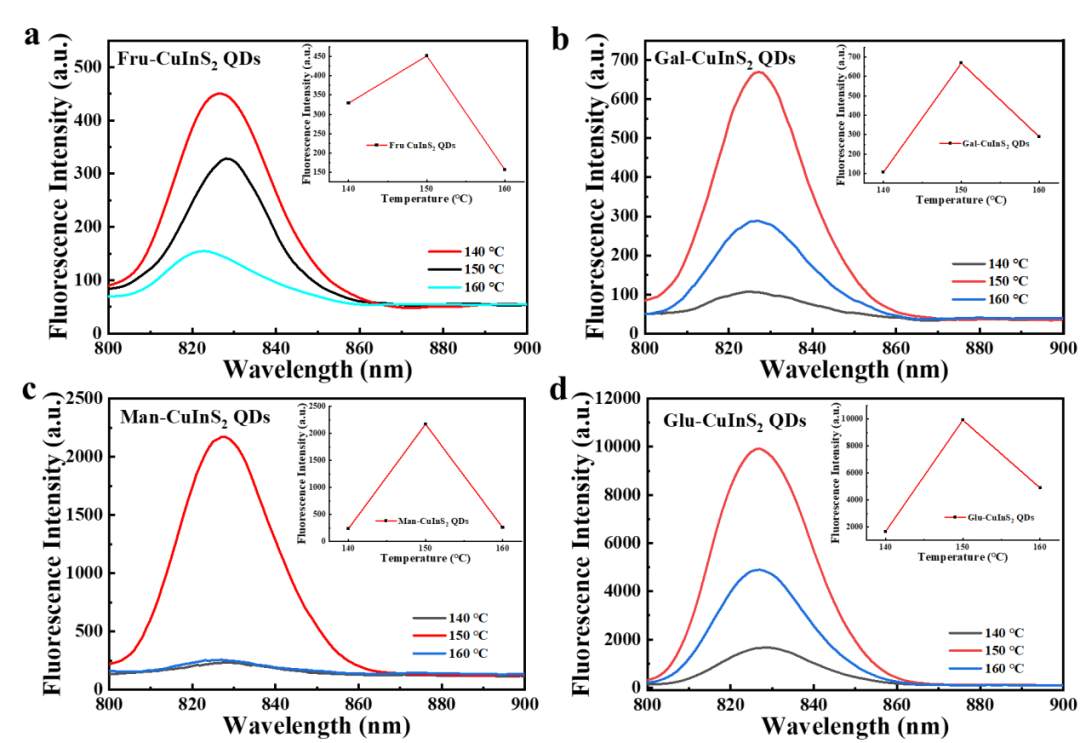


**Figure S5** Fluorescence emission spectra of (a) Fru-CuInS_2_ QDs, (b) Gal-CuInS_2_ QDs, (c) Man-CuInS_2_ QDs, and (d) Glu-CuInS_2_ QDs synthesized under different temperature conditions. (Inset: Relationship between temperature and luminescence intensity of CuInS_2_ QDs).

**Table S1** Exploration of optimal synthesis conditions for glyco-CuInS_2_ QDs.

|  | Cu: In: S: Ligand | pH | Temperature/℃ |
| --- | --- | --- | --- |
| Fru-CuInS_2_ QDs | 1:1:2:36 | 11 | 150 |
| Gal-CuInS_2_ QDs | 1:1:2:12 | 12 |  |
| Man-CuInS_2_ QDs | 1:1:2:36 | 11 |  |
| Glu-CuInS_2_ QDs | 1:1:2:24 | 11 |  |


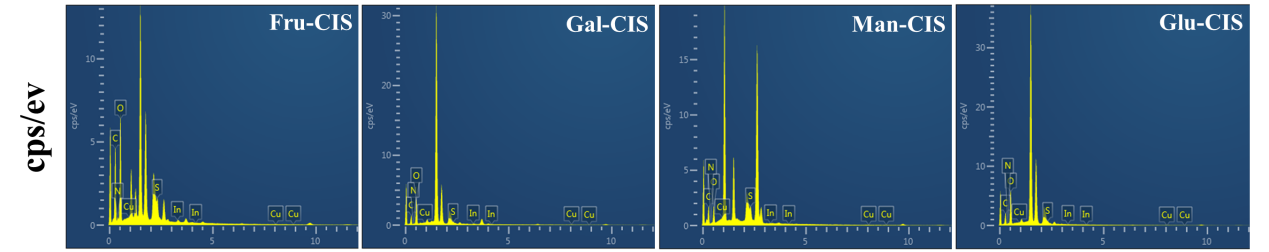


**Figure S6** The SEM-EDS analysis of Fru-CuInS_2_, Gal-CuInS_2_, Man-CuInS_2_ and Glu-CuInS_2_ QDs.


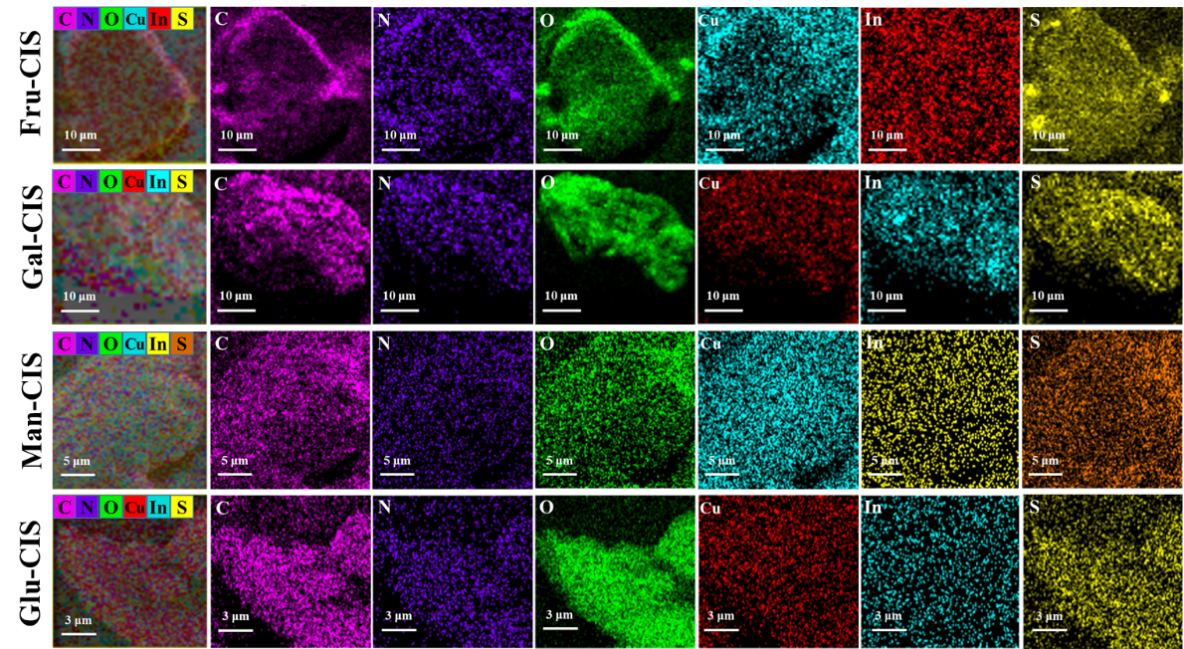


**Figure S7** The EDX elemental mapping of Fru-CuInS_2_, Gal-CuInS_2_, Man-CuInS_2_ and Glu-CuInS_2_ QDs.


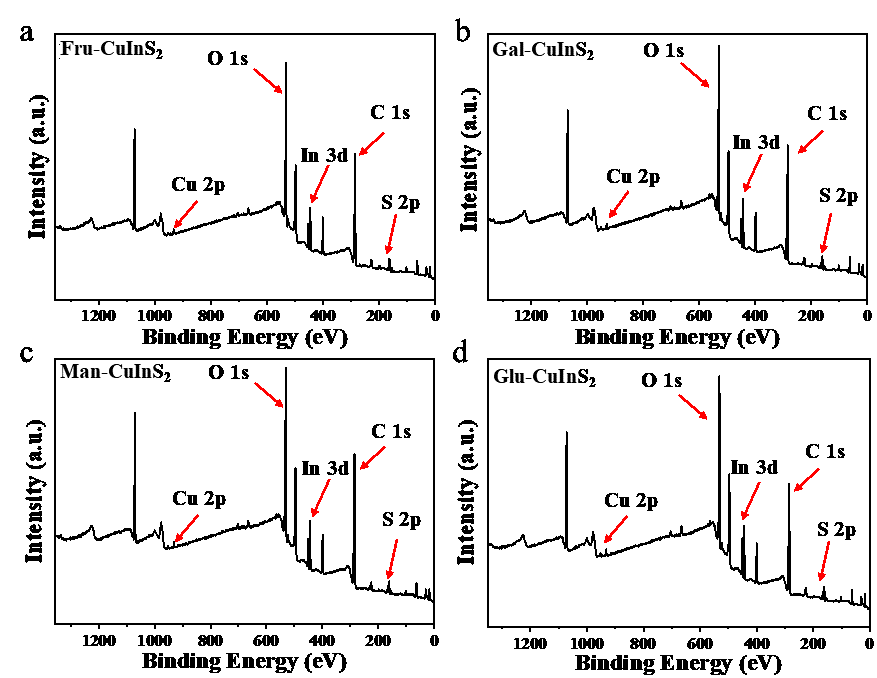


**Figure S8** The XPS survey spectra of (a)Fru-CuInS_2_ QDs, (b)Gal-CuInS_2_ QDs, (c)Man-CuInS_2_ QDs, and (d)Glu-CuInS_2_ QDs.

**Figure S9** Zeta potential values of glyco-CuInS_2_ QDs.


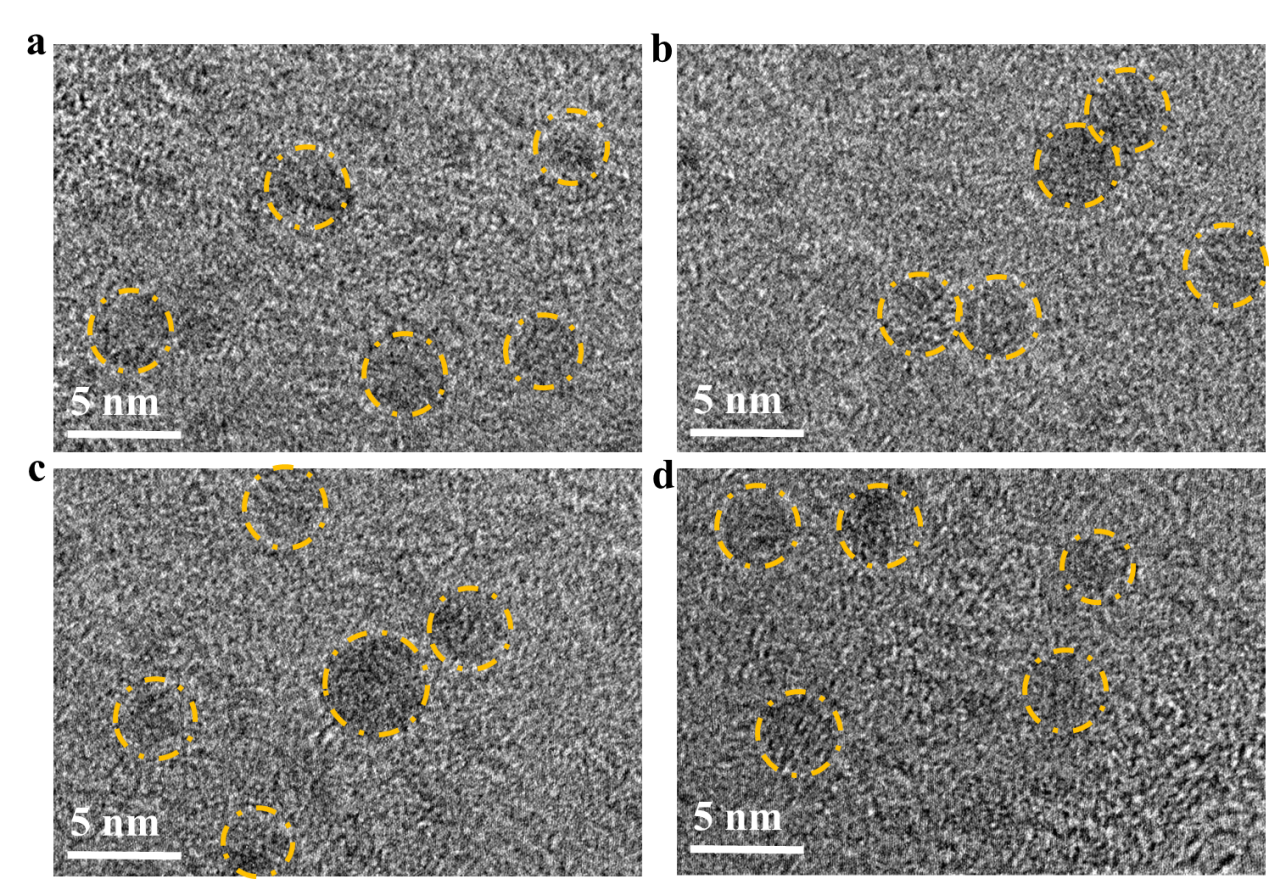


**Figure S10** TEM images of Fru-CuInS_2_, Gal-CuInS_2_, Man-CuInS_2_ and Glu-CuInS_2_ QDs at a scale of 5 nm after fourteen days.


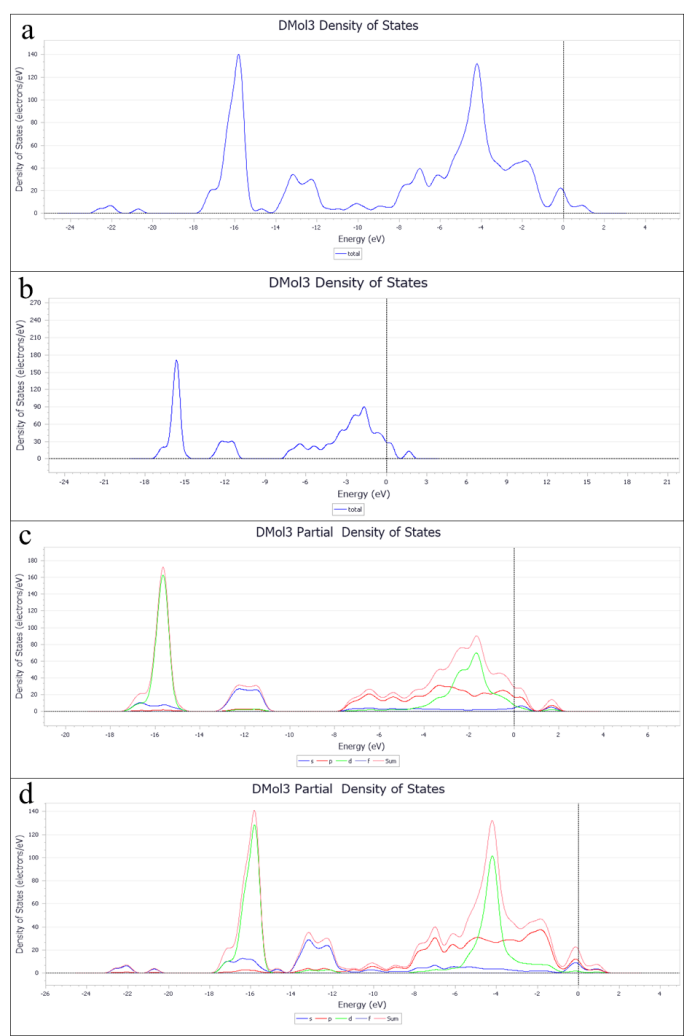


**Figure S11** DOS and PDOS of Fructose molecules adsorbed on CuInS_2_(001) before and after (a) DOS of CuInS_2_(001) system, (b) Fructose molecules adsorbed on DOS of CuInS_2_(001) system, (c) PDOS of CuInS_2_(001) system, (d) Fructose molecules adsorbed on DOS of CuInS_2_(001) system.


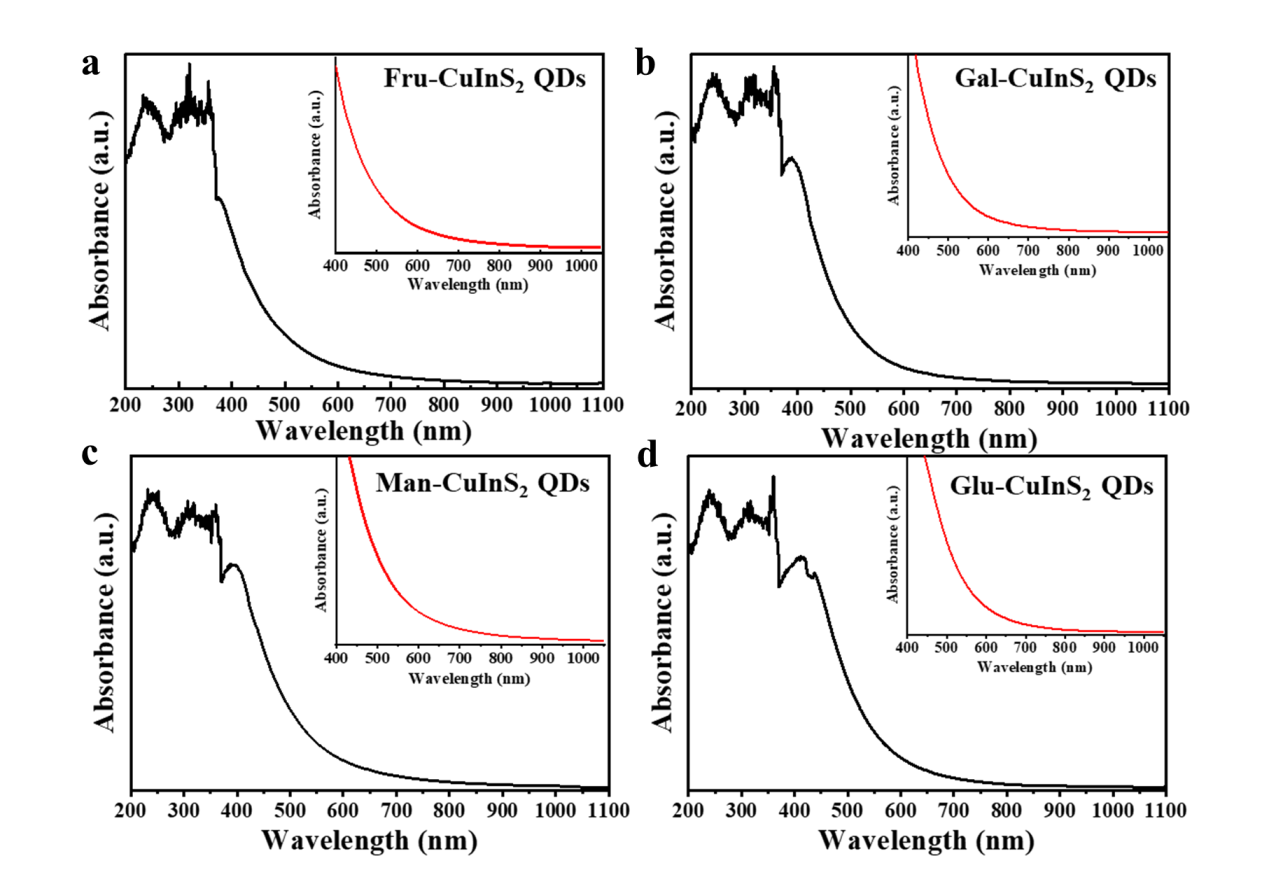


**Figure S12** The UV-Vis absorption spectra of Fru-CuInS_2_, Gal-CuInS_2_, Man-CuInS_2_ and Glu-CuInS_2_ QDs, respectively.

**Table S2** The photophysical properties of glyco-CuInS_2_ QDs.

|  | λ_ex_/nm | λ_em_/nm | τ/ns | χ^2^ | QY/% |
| --- | --- | --- | --- | --- | --- |
| Fru-CuInS_2_ QDs | 515 | 582 | 4.27 | 1.1165 | 15.05 |
| Gal-CuInS_2_ QDs | 415 | 500 | 4.71 | 1.0867 | 10.05 |
| Man-CuInS_2_ QDs | 395 | 502 | 3.96 | 1.2591 | 4.60 |
| Glu-CuInS_2_ QDs | 455 | 553 | 6.61 | 1.0874 | 12.10 |


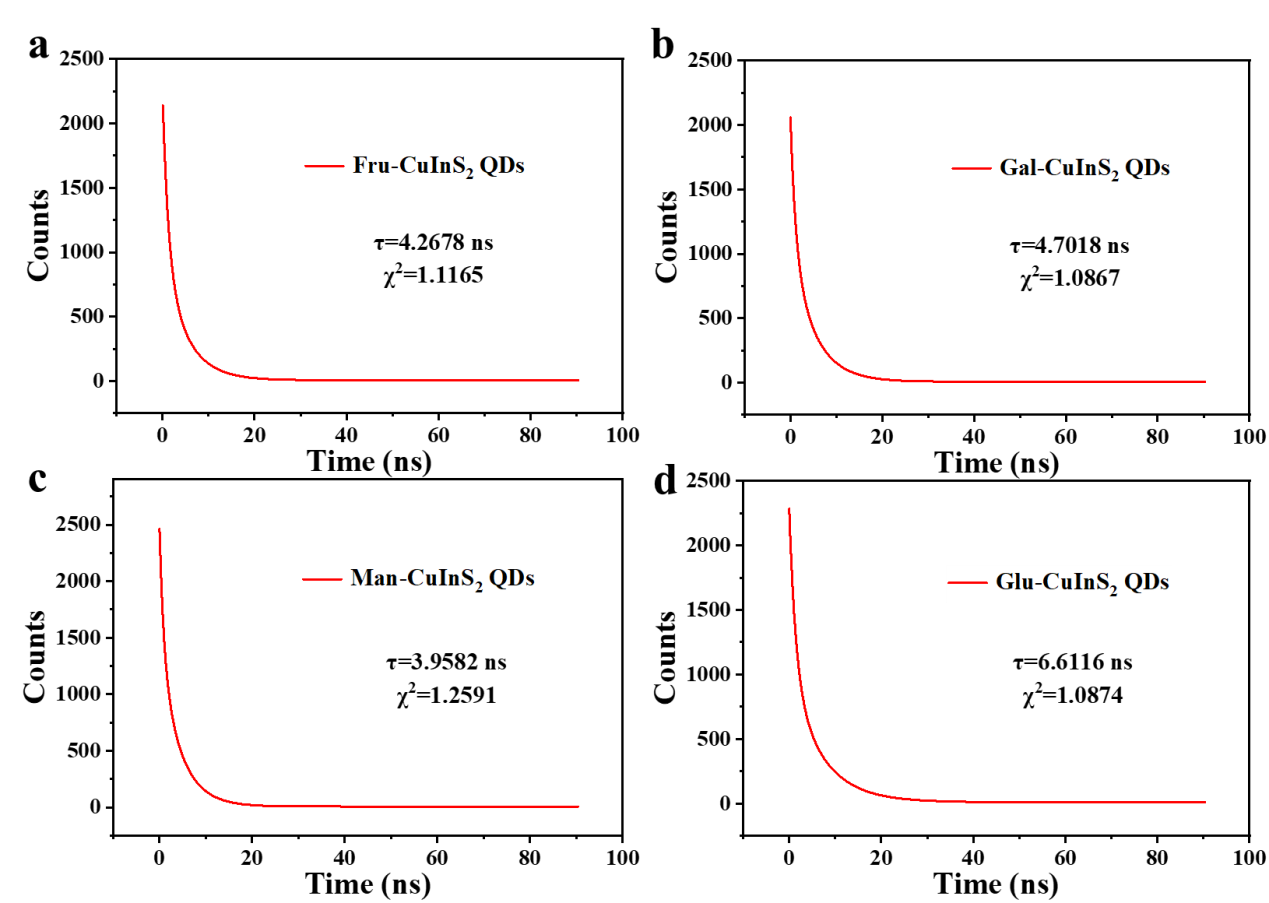


**Figure S13** Fluorescence lifetimes of glyco-CuInS_2_ QDs.


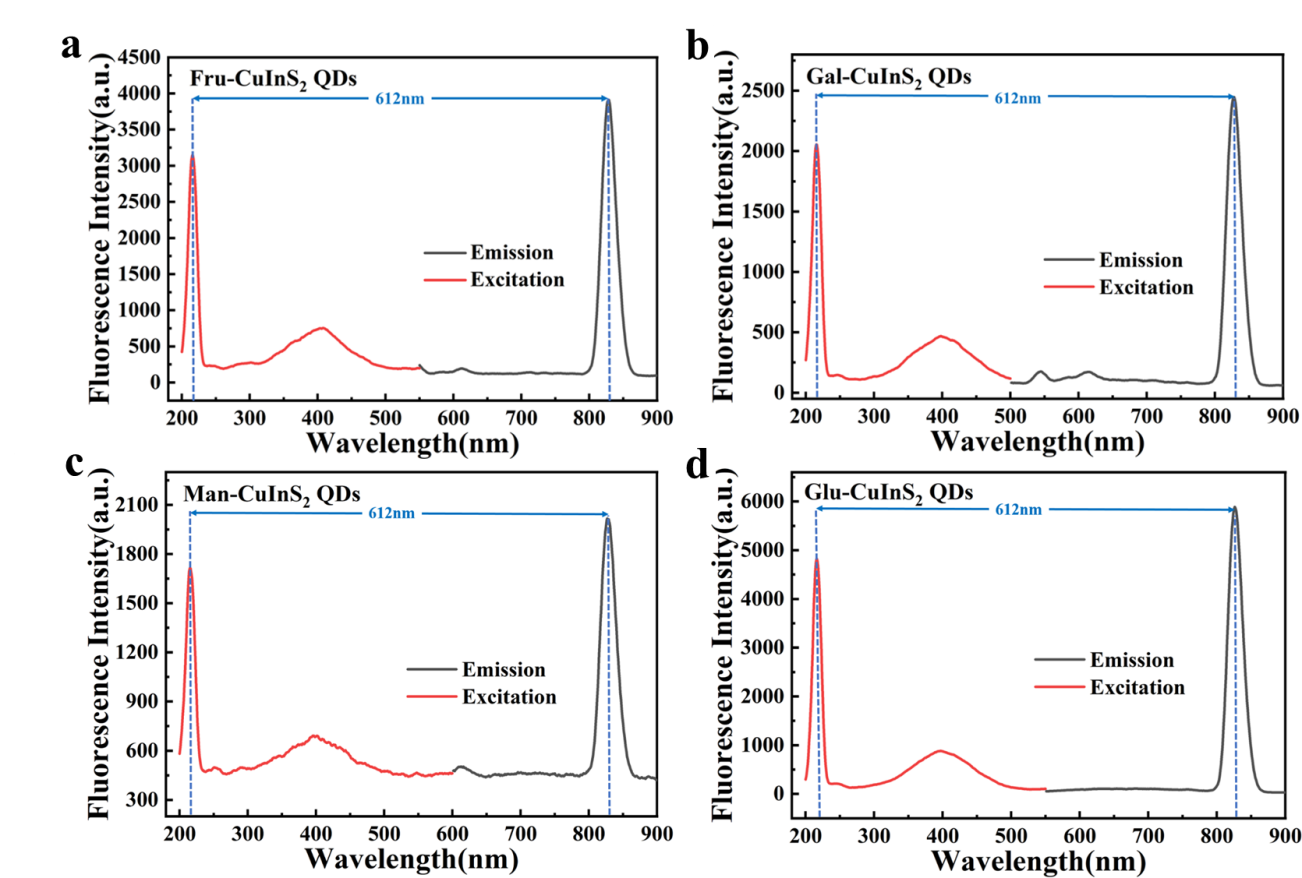


**Figure S14** Excitation and emission fluorescence spectra of (a) Fru-CuInS_2_ QDs, (b) Gal-CuInS_2_ QDs, (c) Man-CuInS_2_ QDs, and (d) Glu-CuInS_2_ QDs.


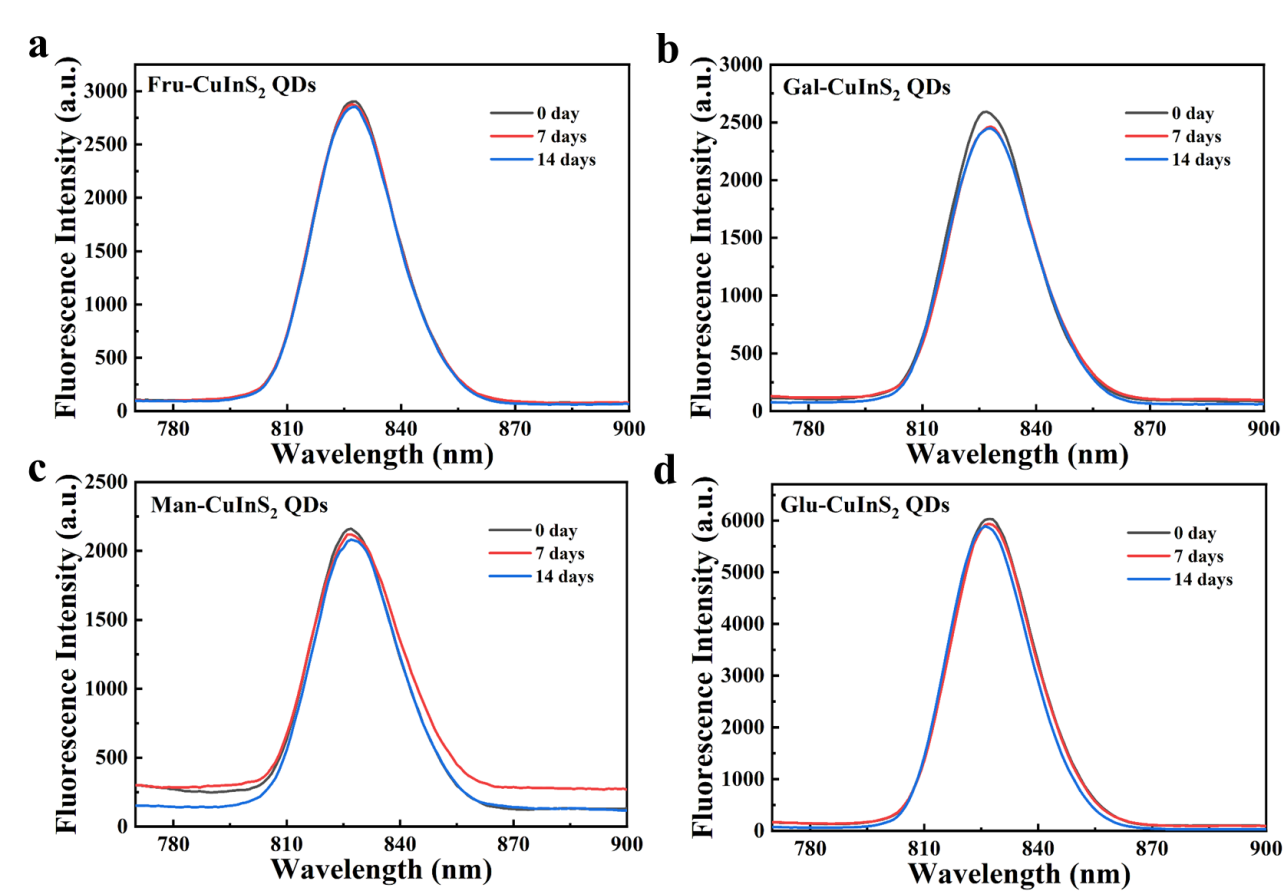


**Figure S15** Fluorescence stabilities of (a) Fru-CuInS_2_ QDs, (b) Gal-CuInS_2_ QDs, (c) Man-CuInS_2_ QDs and (d) Glu-CuInS_2_ QDs in solution. The black, red and blue lines refer to the fluorescence spectra of glyco-CuInS_2_ QDs after 0, 7, and 14 days, respectively.


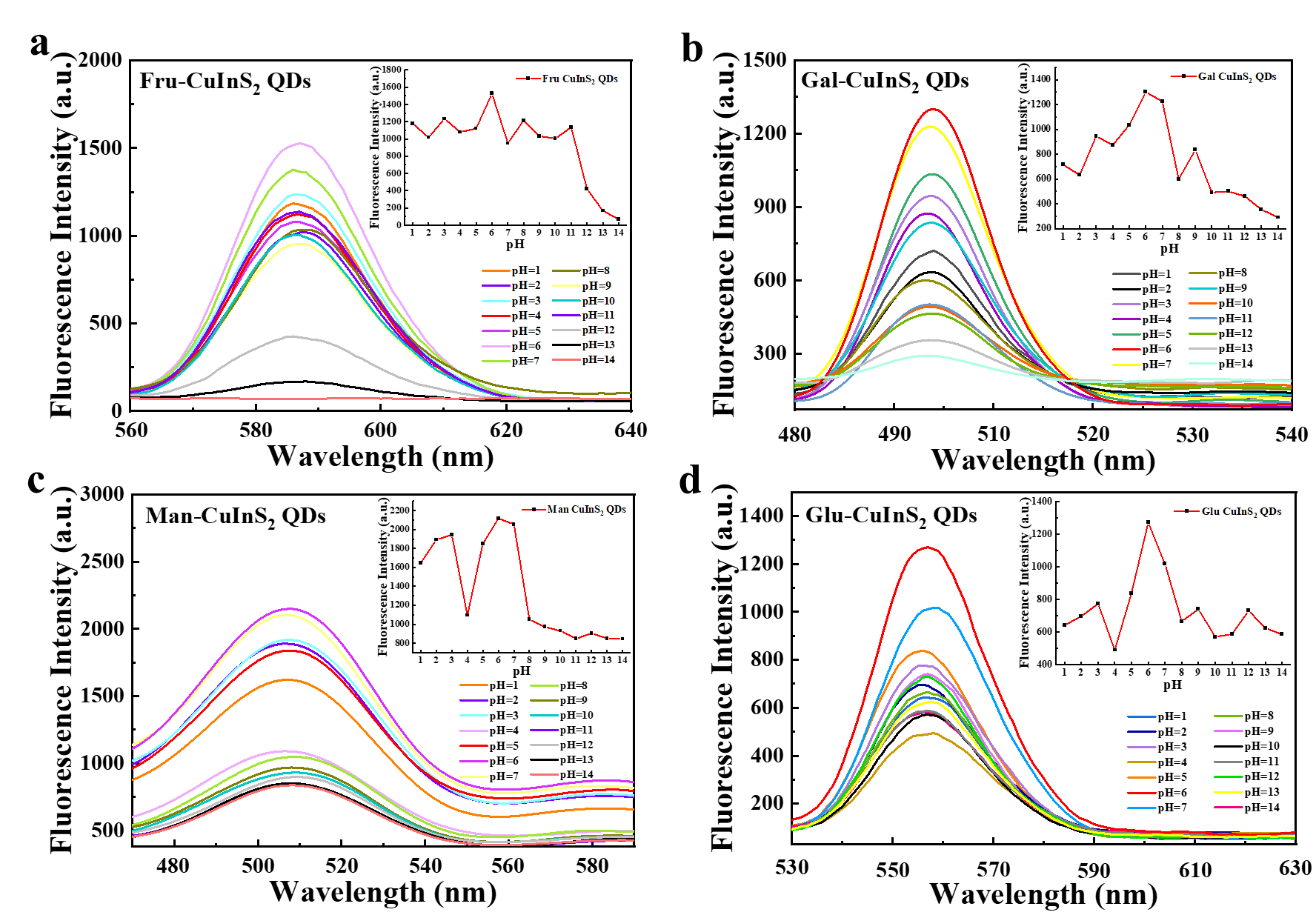


**Figure S16** Fluorescence spectra of the pH response of (a) Fru-CuInS_2_ QDs, (b) Gal-CuInS_2_ QDs, (c) Man-CuInS_2_ QDs, and (d) Glu-CuInS_2_ QDs. (Inset: relationship between pH and luminescence intensity of CuInS_2_ QDs)


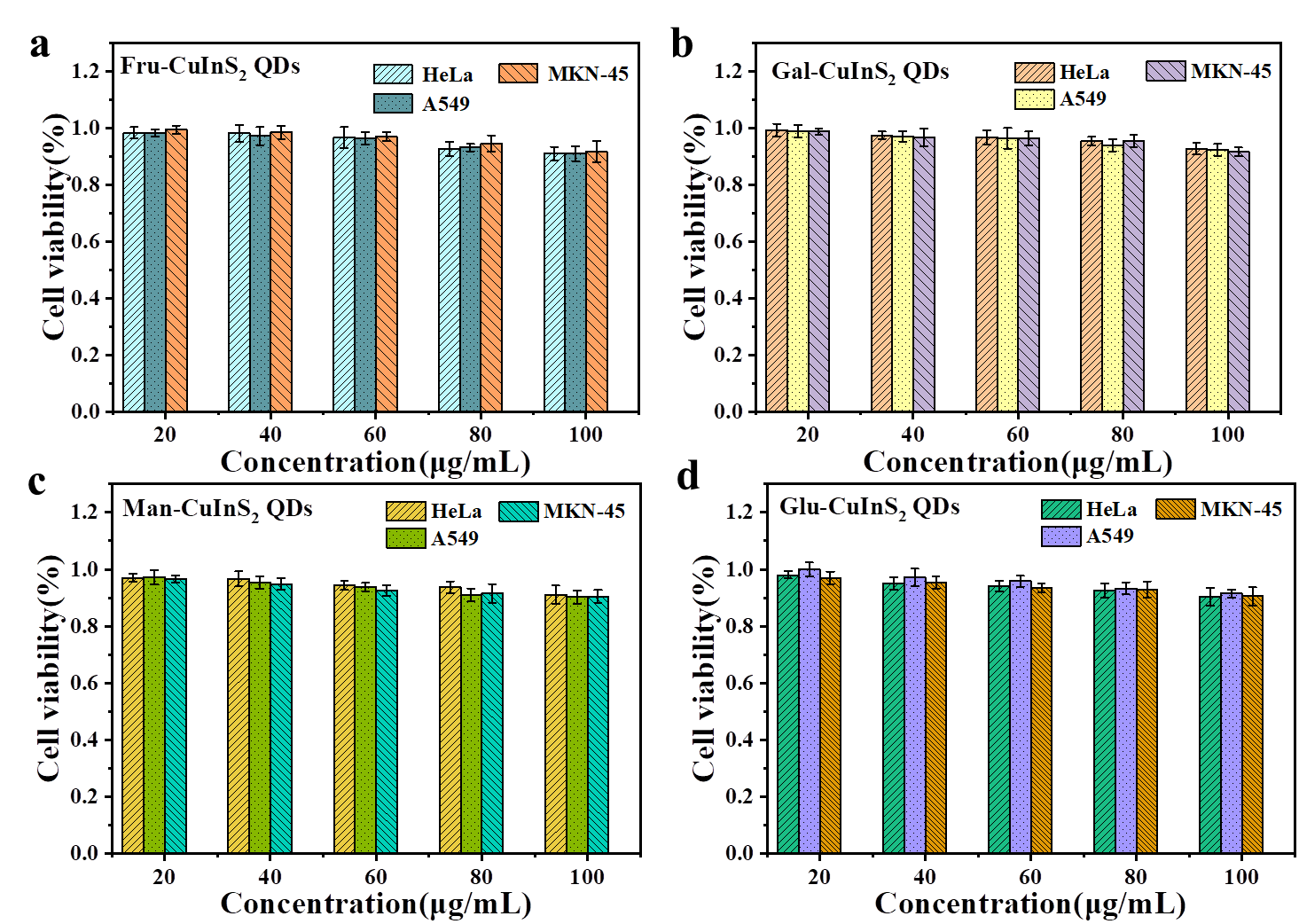


**Figure S17** MTT assay to detect changes in cellular activity of cancer cells after the addition of 20,40,60,80,100 μg/mL of (a) Fru-CuInS_2_ QDs, (b) Gal-CuInS_2_ QDs, (c) Man-CuInS_2_ QDs, and (d) Glu-CuInS_2_ QDs, respectively.

**Figure S18** Fluorescence intensities of (a)Fru-CuInS_2_ QDs, (b) Gal-CuInS_2_ QDs, (c) Man-CuInS_2_ QDs, and(d) Glu-CuInS_2_ QDs imaged under fluorescence microscopy after incubation of zebrafish for 1 h, respectively.

**Video S1-S4** Video of fluorescence performance of glyco-CuInS_2_ QDs in 3D MKN-45 cell microspheres at different times.

**References**

1. S. Liu and X. Su, The synthesis and application of I–III–VI type quantum dots. *RSC Advances* ,2014, **4**, 43415-43428.
2. F. Rasoulzadeh, M. Amjadi and M. Ghorbani, A highly sensitive chemiluminescence assay for diniconazole by using CuInS quantum dots_2_. *Microchemical Journal* ,2020, **159**, 105323.
3. S. Sandra and G.-J. Ilona, Cytotoxicity studies of quantum dots with the electroporation method. *Bioelectrochemistry* ,2018, **126**, 86-91.
4. Y. Ilya, F. Aurelie, G. Maelle, L. Nicolas, F. Alexandra, P. Thomas, B. Lina and M. Frédéric, NIR imaging of the integrin-rich head and neck squamous cell carcinoma using ternary copper indium selenide/zinc sulfide-based quantum dots. *Cancers* ,2020, **12**, 3727.
5. H. Chen, X. He, Y. Yu, Y. Qian, J. Shen and S. Zhao, Execution of aggregation-induced emission as nano-sensors for hypochlorite detection and application for bioimaging in living cells and zebrafish. *Talanta* ,2020, **214**, 120842.
